# Supplementary material for: Exploring the effects of added sugar labels on food purchasing behaviour in Australian parents: An online randomised controlled trial
Source: PLoS One. 2022 Aug 25;17(8):e0271435. doi: 10.1371/journal.pone.0271435 (PMC9409597; doi:10.1371/journal.pone.0271435)
Supplement: S1 File — (DOCX) [file pone.0271435.s001.docx]

**Office use only**

Reference number:

Quiz results received for all applicants? Y/N

| **DEAKIN UNIVERSITY HUMAN ETHICS ADVISORY GROUP**  **LOW-RISK APPLICATION FORM** |  |
| --- | --- |

The [*National Statement on* *Ethical Conduct in Human Research*](https://nhmrc.gov.au/about-us/publications/national-statement-ethical-conduct-human-research-2007-updated-2018) (2007) - updated 2018 defines low risk research as:

‘Research in which the only foreseeable risk is one of discomfort. Research in which the risk for participants

is more serious than discomfort is not low risk’.

**Project Title:** Exploring effectiveness of policy options for added sugar labelling

**Proposed Start Date:** 1/05/2020 **Proposed end date:** 01/01/2022

**Principal Investigator/s:** Miranda Blake

***Please note****: There has been an update by Microsoft which blocks links to single sign-on web pages.*

*To access the Deakin Human Ethics Guidelines referred to in this form, cut and paste the following address to your browser:*

<http://www.deakin.edu.au/students/research/research-support-and-scholarships/integrity-secure/human-ethics/dheg>

*Links to external sites in this form will still work.*

**COVID-19**

Please indicate whether:

1. Your project is not designed to align with current COVID-19 restrictions but will be delayed until all restrictions are lifted and will be modified with approval prior to commencement in the event that unforeseen flow-on effects from the virus raise new ethical issues

OR

1. Your project is designed to align with current COVID-19 restrictions and will be conducted once approval is granted

**PART A: Excluded Categories** (see *National Statement* [Chapter 5.1.6](https://nhmrc.gov.au/about-us/publications/national-statement-ethical-conduct-human-research-2007-updated-2018#guidelines___chapter_5_1))

**1 Does your project involve any of the following?**

Yes  No  Aboriginal or Torres Strait Islander Peoples or issues

Yes  No  Research involving pregnant women or the human fetus

Yes  No  People highly dependent on medical care who may be unable to give consent

Yes  No  People with a cognitive impairment, an intellectual disability, or mental illness

Yes  No  People who may be involved in illegal activities where the research is intended to study or expose illegal activity or that is likely to discover it

Yes  No  Clinical trials - defined as “…*any research study that prospectively assigns human participants or groups of humans to one or more health-related interventions to evaluate the effects on health outcomes”* (World Health Organisation)

Yes  No  Human genomic research

Yes  No  Human biospecimens including human cells (Please note: where researchers want to re-use human biospecimens that were originally collected as per a Deakin approved ethics application, their future use *may* be eligible for low risk review if the researchers intend to seek specific consent from participants for the new project and if all other aspects of the project are low risk)

Yes  No  Projects involving ionising radiation

Yes  No  Travel to regions classified as Level 2, 3 or 4 (see [DFAT](http://smartraveller.gov.au/resources/Pages/travel-advice-explained.aspx) and Section 35 of the Deakin Guidelines)

Yes  No  Projects involving active concealment or planned deception of participants

Yes  No  Risk of harm to participants (more serious than discomfort, *National Statement* [Chapter 2.1.6](https://nhmrc.gov.au/about-us/publications/national-statement-ethical-conduct-human-research-2007-updated-2018#guidelines___chapter_2_1))

Yes  No  Opt-out consent in relation to the collection of ***health or sensitive data** from participants (*National Statement*, [Chapter 2.3](https://nhmrc.gov.au/about-us/publications/national-statement-ethical-conduct-human-research-2007-updated-2018#introduction___chapter_2_3)). Please complete a [Human Research Ethics Application](https://hrea.gov.au/) (HREA) that includes a request to waive the requirement for consent (required under Privacy Legislation) and submit to DUHREC, attaching the [Privacy Supplement](https://www.deakin.edu.au/students/research/research-support-and-scholarships/integrity-secure/human-ethics/human-ethics-forms-and-guidelines/application-forms).

Yes  No  A waiver of the requirement for consent when accessing collections of potentially **identifiable *health or sensitive data^+^**. Please complete a [Human Research Ethics Application](https://hrea.gov.au/) (HREA) and submit to DUHREC attaching the [Privacy Supplement](https://www.deakin.edu.au/students/research/research-support-and-scholarships/integrity-secure/human-ethics/human-ethics-forms-and-guidelines/application-forms). Please note if you are accessing **pre-existing collections of non-identifiable data** you may be eligible for an exemption (see Q.3, page 3 for more information).

**If you selected yes to ANY of these elements, your project is not eligible for low-risk review.** You should complete a higher than low risk application form (the [Human Research Ethics Application](https://hrea.gov.au/) (HREA) for review by DUHREC (see [Section 6.5](https://www.deakin.edu.au/students/research/research-support-and-scholarships/integrity-secure/human-ethics/dheg/g6) of the Deakin Guidelines).

**^+^ Please note:** if you are seeking access to existing collections of **potentially identifiable data**, that are **neither health nor sensitive data**, your project may still be eligible for low risk review even if you are seeking to waive the requirement for consent (i.e. you are not planning to obtain consent from participants to access their data) so long as every other aspect of your project is low risk. If this is the case, please complete Q.1 in Section 2 of the Checklist, and Questions 3 and 11 in Part C of this form. Please also note: this only applies to existing collections of data. If you are prospectively collecting data directly from participants, you will need to apply for ethics approval and seek participant consent prior to collecting the data. If you are using **opt-out consent in relation to the collection of non-health or non-sensitive data**, and every other aspect of your study is low risk, please address the National Statement 2.3.6 a-i (see Part C: Q.10: Consent).

*** Please see Part F: Glossary, page 15 of this form for definitions of health and sensitive data.**

**2 Does your project involve ethical review by another Australian organisation?**

Yes  No  If yes, your project should not be submitted for review by a HEAG. You should consult [Section 6](https://www.deakin.edu.au/students/research/research-support-and-scholarships/integrity-secure/human-ethics/dheg/g6) of the Deakin Guidelines regarding the processes which apply to applications previously approved by another organisation.

**3 Does your project involve ONLY use of existing collections of non-identifiable data?**

Data are non-identifiable when they do not identify the people to whom the information relates – identifiers should never have been collected, or should have been permanently removed from the data set before you received it.

Yes  No  If yes, you should complete the application form for Exemption from Ethical Review (see Section 6 of the Deakin Guidelines). Please note: **research using human biospecimens is not eligible for an exemption.**

**PART B: Checklist**

This checklist will help you decide whether your research may be submitted for review by your Faculty HEAG. Research is eligible for low-risk review if the foreseeable risk level is no more than discomfort. If you answer ‘YES’ to any items on the checklist **your project is not eligible for low risk review** **unless** you can explain how this potential risk will be managed or minimised to ensure that the project remains low risk. This should be explained in the special case assessment section (Section 6) below.

**It is your responsibility to assess the level of risk associated with your project. If your project is not considered low risk by the HEAG, you will be required to complete a high-risk application for submission to DUHREC.**

***Please ensure you include all signatures before submitting the application***

***as approval cannot be granted until they are received.***

# 1 Are any of the following topics to be covered in part or in whole?

| Parenting | YES | NO |
| --- | --- | --- |
| Sensitive personal issues | YES | NO |
| Sensitive cultural issues | YES | NO |
| Grief, death or serious/traumatic loss | YES | NO |
| Gambling | YES | NO |
| Eating disorders | YES | NO |
| Illicit drug taking | YES | NO |
| Substance abuse | YES | NO |
| Self-report of criminal behaviour | YES | NO |
| Any psychological disorder, depression, mood states and/or anxiety | YES | NO |
| Suicide | YES | NO |
| Sexuality, sexual behaviour or gender identity | YES | NO |
| Race or ethnic identity | YES | NO |
| Any disease or health problem | YES | NO |
| Fertility | YES | NO |
| Termination of pregnancy | YES | NO |

# 2 Are any of the following procedures to be employed?

| Waiver of consent for access to collections of identifiable data that are neither health nor sensitive data (please see Part F: Glossary for definitions of health and sensitive data). | YES | NO |
| --- | --- | --- |
| Use of personal data obtained from Commonwealth or State Government Department/Agency | YES | NO |
| Concealing the purposes of the research | YES | NO |
| Covert observation | YES | NO |
| Audio or visual recording without consent | YES | NO |
| Recruitment via a third party or agency | YES | NO |
| Withholding from one group specific treatments or methods of learning, from which they may ‘benefit’(e.g. in medicine or teaching) | YES | NO |
| Psychological interventions or treatments | YES | NO |
| Administration of physical stimulation | YES | NO |
| Invasive physical procedures | YES | NO |
| Infliction of pain | YES | NO |
| Administration of drugs or placebos | YES | NO |
| Administration of other substances | YES | NO |
| Use of medical records where participants can be identified or linked | YES | NO |

#### 3 PARTICIPANT VULNERABILITY ASSESSMENT

**Does the research specifically target participants from any of the following groups?**

| Children or young people under 18 years | YES | NO |
| --- | --- | --- |
| People with a physical disability or vulnerability | YES | NO |
| People whose ability to give consent is impaired | YES | NO |
| Residents of a custodial institution | YES | NO |
| People unable to give free informed consent because of difficulties in understanding the Plain Language Statement or Information Sheet (e.g. language difficulties) | YES | NO |
| Members of a socially identifiable group with special cultural or religious needs or political vulnerabilities | YES | NO |
| People in dependent or unequal relationship with the researchers (e.g. lecturer/student, doctor/patient, teacher/pupil, professional/client) | YES | NO |
| People with existing relationships with the researcher (e.g. relative, friend, co-worker) | YES | NO |
| People in a workplace setting with the potential for coercion or problems of confidentiality (e.g. employer/employee) | YES | NO |
| Participants able to be identified in any final report when specific consent for this has not been given | YES | NO |
| Persons not usually considered vulnerable but would be thought so in the context of the project | YES | NO |

**4. RESEARCH IN OVERSEAS SETTINGS ASSESSMENT**

**Does the research involve any of the following?**

| Research being undertaken in a politically unstable area | | | YES | NO |
| --- | --- | --- | --- | --- |
| Research involving sensitive cultural issues | | | YES | NO |
| Research in countries where criticism of government and institutions might put participants and/or researchers at risk | | | YES | NO |
| *Please indicate which DFAT level of advice applies to the region you intend visiting (see [DFAT](http://smartraveller.gov.au/resources/Pages/travel-advice-explained.aspx) and Section 35 of the Deakin Guidelines) | 1 | 2 | 3 | 4 |
| **Note: Travel to regions classified as Level 2 (or above) is considered higher than low risk and not eligible for review by a HEAG.* | | | | |

## 5. OTHER RISKS

| Are there any risks to the researcher, (e.g. research undertaken in unsafe environments or trouble spots)? | YES | NO |
| --- | --- | --- |
| Are there any other risks not covered in this assessment that you consider may be relevant? | YES | NO |

**6. SPECIAL CASE ASSESSMENT**

If you have answered ‘YES’ to an item in the checklist but you still believe that because of the particular nature of the project and the participants your project may still be eligible for low risk review. Please provide details below, or attach an additional sheet.

**SPECIAL CASE DETAILS:**

A third-party recruitment company will be used for the online study. We have previously used recruitment companies to ensure that participants can be recruited according to required demographic quotas. The recruitment company have security measures in place to securely store participants’ identifiable data. No identifiable information will be received by the researchers from the recruitment company. Research data (i.e. answers to the survey) will not be available to or stored by the recruitment company.

**PART C: Project**

## Aims of the project/the research question(s)/hypothesis

*Aim:*

This project aims to generate experimental evidence from two online RCTs to determine the relative effect of individual and combined display of proposed added sugar labels to facilitate healthier choices by consumers, and to examine relative differences by food product type, and by consumer age, literacy, and levels of sugar consumption. This new evidence will be critical to the development of effective labelling policies to reduce added sugar consumption for all Australians and New Zealanders.

*Research question:*

Primary

What is the impact of added sugar labels on intended purchase of selected high sugar food and drink products?

Secondary

What is the impact of different combinations of added sugar labels on intended purchase of selected high sugar food and drink products?

How does the effect of added sugar labels on intended purchase of selected high sugar food and drink products differ according to participant characteristics (including age, gender, education, income, or usual levels of sugar consumption)?

*Hypothesis:*

Primary

Added sugar labelling on food and drink items reduces the proportion of intended purchases that are high sugar products, compared to no added sugar labels.

Secondary

Displaying multiple added sugar labels on food and drink items reduces the proportion of intended purchases that are high sugar products, compared no labels or display of one added sugar label.

Participant characteristics including age, gender, education, income, and usual levels of sugar consumption alter the effect added sugar labelling on intended purchasing.

## Research design and methods

Give a concise and simple description of the proposed research design and the methods to be used. Please include all data collection procedures and all groups of participants.

The project will consist of two stages of Randomised Clinical Trials (RCT) via an online survey platform. We will work with our online survey recruitment agency (likely Aptigence recruitment). A sample of 1,008 participants aged 18 and over who regularly (at least once a month) purchase yogurt, breakfast cereals and packaged beverages for themselves or household will be recruited for Trial 1. Participants will be contacted directly by the recruitment company and invited to participate in the study. They will then be directed to the survey weblink, which opens with the PICF. Eligible participants who agree to participate will be randomised to complete an anonymous online survey consisting of three hypothetical purchasing tasks: one each for breakfast cereals, yoghurts and packaged beverages. Each task will consist of participants selecting between ten alternative products with varying sugar content, labelled with one of seven labelling options currently used in Australia or internationally or under consideration by the Australian Government, for example (but not limited to): (i) Status quo – current Nutrition Information Panel (Back of Pack labelling system without added sugar content) (control condition); (ii) Pictorial warning label; (iii) Teaspoons of Sugar; (iv) Textual warning Label (Appendix 1: Sample survey using control and Textual warning label); see Figure 1 for label options). Participants will then be asked to select which item they would most likely purchase from a variety of options.

| Label 1: Status quo – current Nutrition Information Panel (NIP) (Back of Pack labelling system without added sugar content) (control condition)  Label 2: Added sugars quantified in the NIP  Label 3: Pictorial approaches to convey the amount or types of sugars in a serving of food  Label 4: Chilean-style advisory labels for foods high in added sugars;  Label 5 and 6: Health Star Rating  Label 7: Change to statement of ingredients to asterisk ingredients with added sugar | 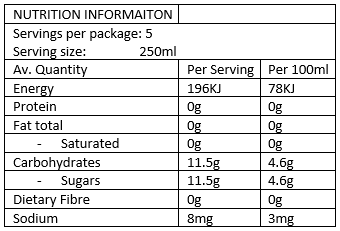  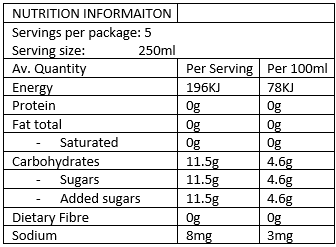  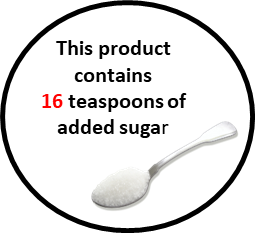  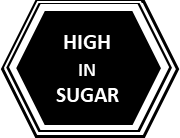  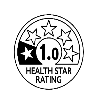  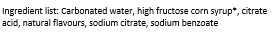 |
| --- | --- |

Figure 1: Examples of intervention labels 1-7 used in the choice experiment study. Health Star Rating trademarks are owned by the Commonwealth of Australia. Further information on the Health Star Rating can be found at www.healthstarrating.gov.au

The seven labelling options in Trial 1 will be distinct, these seven distinct labels will be combined to determine the effect of displaying multiple labels and effects for different consumer subgroups. The two most effective labels from Trial 1 will be used to determine four test scenarios for Trial 2: Label A; Label B; Label A+B; Status quo (control condition). 1,152 Trial 2 participants will be recruited to cover a spectrum of ages in the Australian population.

Sample size calculations: Sample sizes of 144 participants per subgroup for Trials 1 and 2 are based on detection of 32% difference in purchasing of high versus low sugar products, with a statistical significance level of 5% and 80% power (based on Billich et al. 2018 findings, smallest difference detected between baseline purchases [64% purchased sugar sweetened beverages] compared to 28% to 47% participants across treatment groups, and approximately 10% unusable surveys due to errors). Trial 1: 7 treatment groups x 144 participants per group= 1,008 participants. Trial 2: 4 treatment groups x 144 participants per group x 2 for subgroup analysis of high v. low sugar consumers= total 1152 participants).

Participants in both surveys will also complete a series of sociodemographic questions including age, income and consumption of target foods (survey attached).

Describe how the design and methods of the project will enable adequate exploration of the research questions and achieve the aims of the research.

This between-subject online randomised control trial was chosen to allow exploration of the effects of food labels that are not yet available in the real world. The design also allows participant groups to be exposed to a number of different scenarios, whereby different labels will be placed on different food items to determine how these labels impact customer choice.

Describe how the design of the project will maintain respect for participants.

Respect for participants will be maintained through the design of the project. Only relevant questions asked throughout the survey, avoidance of potentially personally sensitive questions, and no collection of identifying information, so participants’ responses will remain anonymous.

Has the project been reviewed by a formally constituted academic, scientific or professional review process and if so, what was the outcome of that review?

Not applicable

## Use of existing stored data

Please list any existing stored data that you plan to use as part of the project e.g. health or employment records used for recruitment, or comparison. Please include in your answer:

- The type and number of records being accessed;
- Whether the records identify individual people; and
- How you will obtain permission to use them (consent from individuals or permission from custodians of non-identifiable data). Or, if not seeking consent, please indicate here that you are seeking a waiver of the requirement for consent and answer Q.11 below (please note waivers of consent can only be sought via low risk review **where the data are neither health nor sensitive data**).

(See the *National Statement* [Chapter 3.1](https://nhmrc.gov.au/about-us/publications/national-statement-ethical-conduct-human-research-2007-updated-2018#guidelines___chapter_3_1) and [Chapter 3.2](https://nhmrc.gov.au/about-us/publications/national-statement-ethical-conduct-human-research-2007-updated-2018#introduction___chapter_3_2); and [Section 25](https://www.deakin.edu.au/students/research/research-support-and-scholarships/integrity-secure/human-ethics/dheg/g25) of the Deakin Guidelines for more information.)

Existing stored data will not be used

Please describe any stored biospecimens that you intend to use as part of the project. **Please note:** projects involving biospecimens may only be considered low risk if they were collected as part of a project that was approved by a Deakin ethics review body, you intend to seek the consent of the participants for the re-use of their biospecimens as described in this application and the project is low risk in all other respects.

(see the *National Statement* [paragraphs 3.2.11-3.2.14](https://nhmrc.gov.au/about-us/publications/national-statement-ethical-conduct-human-research-2007-updated-2018#chapter_3_2__human_biospecimens_in_laboratory_based_research) for more information)

Not applicable

## Risks and benefits

**Give a summary of the expected benefits of this project**

This may include benefits to the broader community, the participants, people with whom the participants identify or the researcher (see the *National Statement* on [benefits](https://nhmrc.gov.au/about-us/publications/national-statement-ethical-conduct-human-research-2007-updated-2018#section_2__themes_in_research_ethics__risk_and_benefit__consent) for more information). Among other things, benefits may include a contribution to knowledge or understanding, improved social or individual wellbeing, or the skill and expertise of researchers.

The Australian and New Zealand Governments are currently exploring options for labelling of added sugars on packaged foods and drinks, to support consumers to reduce their sugar intake. The possible benefits will be disseminated through the Obesity Policy Coalition networks (by Associate Investigator Dr Martin) including directly to policy makers to inform current policy consultations. The effectiveness and feasibility of implementing front-of-pack or point-of-sale signage addressing the sugar content on specific food items will also be disseminated to relevant parties at government agencies including Food Regulation Standing Committee (FRPC), Australian Department of Health, and the New Zealand Ministry for Primary Industries (by Associate Investigator CN Mhurchu). This in turn will provide evidence to inform policy aimed at preventing obesity and chronic disease.

**Provide a justification for the potential benefits**

Potential benefits should be based on either relevant literature or a review of prior research unless, due to the novelty of the question, there is scarce literature or prior research.

“Added” sugars include sugar added to foods and beverages in the production or cooking process(1) . “Free” sugars are a broader category of sugars, and include “all monosaccharides and disaccharides added to foods by the manufacturer, cook, or consumer, plus sugars naturally present in honey, syrups, and fruit juices and fruit juice concentrate” except those that are naturally occurring or present in foods such as fruits, vegetables and milk (2). Over half of Australians (52%) exceed the World Health Organization (WHO)’s recommended intake of added sugar (3), increasing their risk of obesity, Type 2 Diabetes, dental decay, and some cancers. Further, the majority (90%) of Australians also exceeded the WHO conditional recommendation that added sugars be reduced to less than 5% of energy intake, where additional health benefits have been shown (3). Food in Australia and NZ is not currently required to display added sugar content, either on front-of-package or in the nutrition information panel on the back of pack. This is an area of strong public and policy interest internationally. There is moderate to strong evidence from laboratory trials that front-of-package added sugar warning labels reduce intended purchasing of sugar-sweetened beverages(4). This includes evidence from our previous online randomised control trial (RCT) which demonstrated that front-of-package labels, including graphic warning labels, reduced intention to purchase sugar-sweetened beverages among Australians aged 18-35 years(5). However, there is limited evidence of labelling effects on actual or intended food purchases for foods other than sugar-sweetened beverages. The Australian and New Zealand Government Food Regulation Standing Committee (FRSC) is exploring options for labelling of added sugars on packaged foods and drinks available for sale in Australia and New Zealand, to support consumers in making informed choices to reduce their added sugar intake. This study will assist with providing relevant evidence to inform policy in this area.

**Give a summary of the expected risks of this project and how they will be managed**

This should include any risks to participants, researchers, to the environment or to Deakin or other organisations (see the *National Statement* on [assessment of risk](https://nhmrc.gov.au/about-us/publications/national-statement-ethical-conduct-human-research-2007-updated-2018#section_2__themes_in_research_ethics__risk_and_benefit__consent) for more information).

We do not foresee any risks to participants. Participants’ names or any other identifiable data will not be received from the recruiting company for the online study.

## Monitoring

As the researcher, how will you monitor the progress of the research?

You should include details of planned communication between members of the research team (e.g. face to face meetings, email, telephone or Skype) (see the *National Statement* [Chapter 5.5.3](https://nhmrc.gov.au/about-us/publications/national-statement-ethical-conduct-human-research-2007-updated-2018#introduction___chapter_5_5http://www.nhmrc.gov.au/book/chapter-5-5-monitoring-approved-research) for more information).

This research will be overseen by Dr Miranda Blake and regular face-to-face and/or tele-conference meetings will be held with the research team. The Research Officer (Devorah Riesenberg) will undertake progress monitoring, through regular telephone and email communication with Dr Miranda Blake, at least weekly through the project.

## Resources

Please explain the amount and source of funding (sponsorship, tender, grant etc.). If there are specific resources required for the project how will they be provided?

This research is being funded by a Faculty HAtCH Grant for 2020. In particular, this funding is for recruitment through a recruitment agency and research fellow time from Ms Riesenberg. Investigators are providing time in kind to the project. No specific resources, outside of those that are already being provided are required for this project.

## Conflict of interest

Do any of the researchers or others involved in this project have any conflict of interest in relation to it? If so, please explain how this will be managed (see the *National Statement* on [Conflict of Interest](https://nhmrc.gov.au/about-us/publications/national-statement-ethical-conduct-human-research-2007-updated-2018#chapter_5_4__conflicts_of_interest) for more information).

No conflicts of interest to declare.

**PARTICIPANTS**

## Describe your participant group/s

Please include the following information for each participant group how many participants you plan to recruit:

- a justification for the number of participants chosen for each participant group
- the inclusion and exclusion criteria.

(See the *National Statement* [Section 4](https://nhmrc.gov.au/about-us/publications/national-statement-ethical-conduct-human-research-2007-updated-2018#section_4__ethical_considerations_specific_to_participants) for more information.)

For the project, we will aim to recruit a total of 2,160 participants (Trial 1 n= 1,008; Trial 2 n= 1,152), each trial will consist of a distinct sample. This sample size was calculated from a power calculation based on a previous study conducted by a member of the current research team

Sample size calculations: Sample sizes of 144 participants per subgroup for Trials 1 and 2 are based on detection of 32% difference in purchasing of high versus low sugar products, with a statistical significance level of 5% and 80% power [based on Billich et al. 2018 findings, smallest difference detected between baseline purchases (64% purchased sugar sweetened beverages) compared to 28% to 47% participants across treatment groups, and approximately 10% unusable surveys due to errors] (5). Trial 1: 7 treatment groups x 144 participants per group= 1,008 participants. Trial 2: 4 treatment groups x 144 participants per group x 2 for subgroup analysis of high v. low sugar consumers= total 1152 participants).

The inclusion criteria for both trials is:

- Australian residents over the age of 18 years
- Access to a computer and internet connection to complete the survey
- Regular (at least once a month or more) purchasers of breakfast cereal, yoghurt and non- alcoholic pre-packaged drinks for themselves or their household
- Completes at least one supermarket shop per month for their household
- Lives with at least one child <18 years at home

## Explain your recruitment process

Please include the following information for each participant group:

- How will you locate the participants that you plan to recruit? If through existing records or contact lists, please explain how this will be done in a way that does not infringe privacy requirements.
- How will initial contact be made?
- If you plan to use a document or spoken statement e.g. flyer, letter, advertisement, phone call, please attach a copy of the document or script to this application.
- All advertisements (both written and spoken) must include the following statement: “This study has received Deakin University ethics approval (reference number: insert reference number here).”
- Will the participants be screened?
- If there is a screening tool, please attach a copy.

(See [Section 8](https://www.deakin.edu.au/students/research/research-support-and-scholarships/integrity-secure/human-ethics/dheg/g8) of the Deakin Guidelines for more information.)

Participants will be recruited by an external recruiting company (likely Aptigence recruitment). The recruitment company owns a database of Australians interested in participating in online surveys. Participants will be contacted by the recruiting company directly and invited to complete the survey through the weblink provided. See online Plain Language Statement (Appendix 2) sheet attached.

Participants will be screened according to the inclusion and criteria in Q8 above. We will also apply the following quota:

- At least 50% of consumer will be considered high sugar consumers. To categorise participants into high sugar purchasers and low sugar purchasers they will be asked to select all relevant products they purchase at least once a month for themselves or their household (related to yoghurt, breakfast cereal and non-alcoholic packaged drinks) out of the 10 options contained in the choice sets. If the participants select one or more high sugar products in each category they will be considered a high sugar consumer

**CONSENT**

## Describe the consent process

There are a variety of ways in which consent can be established, most commonly by giving participants a Plain Language Statement and Consent Form (PLSCF) or by return of a survey. You may wish to consult [Section 9](https://www.deakin.edu.au/students/research/research-support-and-scholarships/integrity-secure/human-ethics/dheg/g9) of the Deakin Guidelines for more information. Please include details such as:

- how and when you will provide consent materials to your potential participants
- how, when and to whom participants will indicate their consent
- If you are seeking **opt-out consent in relation to non-health or non-sensitive data**, please address the National Statement [Chapter 2.3](https://www.nhmrc.gov.au/about-us/publications/national-statement-ethical-conduct-human-research-2007-updated-2018#toc__296), 2.3.6 a-i.

(See the *National Statement* [Chapter 2.2](https://nhmrc.gov.au/about-us/publications/national-statement-ethical-conduct-human-research-2007-updated-2018#chapter_2_2__general_requirements_for_consent), [Chapter 2.3](https://nhmrc.gov.au/about-us/publications/national-statement-ethical-conduct-human-research-2007-updated-2018#chapter_2_3__qualifying_or_waiving_conditions_for_consent) and Element 3 of [Chapter 3.1](https://nhmrc.gov.au/about-us/publications/national-statement-ethical-conduct-human-research-2007-updated-2018#chapter_3_1__the_elements_of_research) for more information.)

An online Plain Language Statement (Appendix 2) describing the nature of the survey will be shown immediately when potential participants invited by the recruitment company click the weblink provided. Participants will click the online consent form to state that they have read the plain language statement and agree to participate.

## Waiver of Consent

Are you seeking a waiver of consent (in relation to accessing **collections of potentially identifiable data** that are **neither health nor sensitive*** data)? (See the *National Statement*, [Chapter 2.3](https://nhmrc.gov.au/about-us/publications/national-statement-ethical-conduct-human-research-2007-updated-2018#chapter_2_3__qualifying_or_waiving_conditions_for_consent) for more information.)  YES  NO

If yes, please describe how this complies with the [NS 2.3.10 a-i](https://www.nhmrc.gov.au/about-us/publications/national-statement-ethical-conduct-human-research-2007-updated-2018#toc__296) requirements below:

1. involvement in the research carries no more than low risk to participants:

1. the benefits from the research justify any risks of harm associated with not seeking consent:

1. it is impracticable to obtain consent (for example due to the quantity, age or accessibility of records):

1. there is no known or likely reason for thinking participants would not have consented if they had been asked:

1. there is sufficient protection of their privacy:

1. there is an adequate plan to protect the confidentiality of data:

1. in case the results have significance for the participants’ welfare there is, where practicable, a plan for making information arising from the research available to them:

1. the possibility of commercial exploitation of derivatives of the data or tissue*(please note waivers of consent in regards to accessing *human tissue or biospecimens are not eligible for low risk review) will not deprive the participants of any financial benefits to which they would be entitled:

1. the waiver is not prohibited by State, federal or international law:

*** Please see Part F: Glossary, page 15 of this form for definitions of health and sensitive data.**

## Will there be reimbursement of expenses or incentives to participate?

Where expenses will be reimbursed please state:

- the nature of the expenses incurred by participants
- the maximum value of any intended reimbursement.

Where incentives to participate are offered, please explain:

- why you consider that the proposed incentive will not encourage participants to take risks they would not otherwise take. In doing so, please consider both the risks associated with participation and the value of the incentive, relative to your participant group.

(See the *National Statement* [Chapter 2.2.10-2.2.11](https://nhmrc.gov.au/about-us/publications/national-statement-ethical-conduct-human-research-2007-updated-2018#chapter_2_2__general_requirements_for_consent); and [Section 8](https://www.deakin.edu.au/students/research/research-support-and-scholarships/integrity-secure/human-ethics/dheg/g8) of the Deakin Guidelines for more information.)

For the online study, participants will receive reimbursement for their time directly from the recruiting company (Aptigence recruitment). The expected reimbursement for each participant is approximately $5. The exact amount is determined by the recruitment agency. Due to its low value, the incentive it is not considered undue inducement or likely be associated with any risks.

Participants are not expected to face any expenses associated with participation.

## Pre-existing or unequal relationships

Do any of the proposed participants have existing relationships with the researchers, each other or with any other organisation involved in the research? Please explain the relationships, and how you will make sure that participants do not feel pressured to take part.

(See the *National Statement* [Chapter 4.3](https://nhmrc.gov.au/about-us/publications/national-statement-ethical-conduct-human-research-2007-updated-2018#chapter_4_3__people_in_dependent_or_unequal_relationships); and the [Section 22](https://www.deakin.edu.au/students/research/research-support-and-scholarships/integrity-secure/human-ethics/dheg/g22) of the Deakin Guidelines for more information.)

No pre-existing or unequal relationships to declare.

## Does your project include children or young people under 18 years?

If your project involves people under the age of 18, please answer the following questions.

- What age group is involved?
- Will parental/guardian consent be obtained? If the young people will consent on their own behalf, how their capacity to do this will be judged?
- Is it necessary to involve people under 18? Could your projects be undertaken with adult participants?
- Is the methodology appropriate for children/young people?
- Is there any reason to consider that participation in the research is not in the best interests of the children/young people?

(For further information, consult the *National Statement* [Chapter 4.2](https://nhmrc.gov.au/about-us/publications/national-statement-ethical-conduct-human-research-2007-updated-2018#chapter_4_2__children_and_young_people); and [Section 19](https://www.deakin.edu.au/students/research/research-support-and-scholarships/integrity-secure/human-ethics/dheg/g19) of the Deakin Guidelines.)

No, the project does not include children or young people under 18 years of age.

## Language and communication issues

Will your project involve people who cannot communicate easily in English? (e.g. people who are not confident English speakers, or who have a disability, such as a hearing impairment that requires special arrangements for participation). If so, please explain how translation/interpretation issues will be managed.

(For further information consult [Section 24](https://www.deakin.edu.au/students/research/research-support-and-scholarships/integrity-secure/human-ethics/dheg/g24) of the Deakin Guidelines.)

No, the project will not involve people who cannot communicate easily in English.

## People in other countries

If you are planning to undertake research in other countries, please answer the following questions. What are the legal and ethical requirements for conducting research in the designated country?

- What arrangements will be in place for a local, readily accessible contact to receive responses, questions and complaints about the research? (*National Statement* [Chapter 4.8.16](https://nhmrc.gov.au/about-us/publications/national-statement-ethical-conduct-human-research-2007-updated-2018#chapter_4_8__people_in_other_countries))
- How will the research be monitored on site?
- Are there cultural sensitivities relating to the research? How will these be managed?

If the research is to be conducted in a language other than English, please ensure that you have covered all relevant language issues under question 14.

(For further information consult the *National Statement*, [Chapter 4.8](https://nhmrc.gov.au/about-us/publications/national-statement-ethical-conduct-human-research-2007-updated-2018#chapter_4_8__people_in_other_countries); and Section 35 of the Deakin Guidelines.)

No, the project will not take place in any country other than Australia.

1. **Return of research results or findings to participants**

If the results/findings of the research will be returned to participants, please explain:

- If the individual research results for each participant will be returned to that participant
- If the overall research findings will be returned to participants
- How these results/findings will be provided to participants/how the process will be managed and
- Any risks associated with returning the results/findings

Participants will be provided with the opportunity to contact the researchers directly to request a lay summary of the results, when available (see PICF). This information will be collected separately to the survey so that participant responses cannot be associated with their personal details. These results will be emailed to interested participants after the publication of the study, approximately end June 2021. Individual-level results cannot be provided as participation is anonymous; all results will be summarised across participant responses. There are no risks anticipated with returning the results/findings.

Where the results/findings could have significant health, social, economic, legal, psychological or other implications for participants or their relatives, please provide an ethically defensible plan to disclose or withhold results or findings of the research. The plan should include:

- What results will be returned (if any)
- Whether the participants will be advised in advance of the option to receive the findings or results
- Whether the findings or results may be given to anyone else and if so, whether participants will be informed of this in advance
- If applicable, the process for determining whether participants’ relatives wish to receive the findings or results
- How the findings or results will be returned in a manner that is appropriate and accessible
- The relevant expertise of the person who may be communicating the results and
- Any measures to protect the level of privacy desired by participants.

Not Applicable

For further information, consult the National Statement paragraphs [3.1.63 - 3.1.65](https://nhmrc.gov.au/about-us/publications/national-statement-ethical-conduct-human-research-2007-updated-2018#chapter_3_1__the_elements_of_research).

Where the research involves the use of biospecimens (see exception to excluded categories of research in Section A), please also include the additional details for the ethically defensible plan described in the National Statement [3.2.15](https://nhmrc.gov.au/about-us/publications/national-statement-ethical-conduct-human-research-2007-updated-2018#chapter_3_2__human_biospecimens_in_laboratory_based_research) points a-i:

Not Applicable

**CONFIDENTIALITY / PRIVACY**

## Will you be collecting data/information in identified form?

Data are generally divided into:

- **identifiable** (also called personal): the person to whom the data relates can be established from the data – either because they are named, or information that identifies them is included (e.g. position in an organisation at the time)
- **re-identifiable** (also called coded): the identifiers have been removed from the information and replaced with a code.
- **non-identifiable**: the data were collected anonymously, or all identifiers have been permanently removed.

Please explain the form in which the data will be collected. If you plan to collect it in identified form and later remove the identifiers, please explain how and when.

(See Section 10 of the Deakin Guidelines for more information.)

Non-identifiable.

## How will the research comply with relevant regulations or guidelines authorised by law?

For example, the mandatory reporting requirements for disclosure of child abuse. For more information see National Statement [3.1.66 - 3.1.68](https://nhmrc.gov.au/about-us/publications/national-statement-ethical-conduct-human-research-2007-updated-2018#chapter_3_1__the_elements_of_research).

It is not expected that these issues will arise. However, all researchers will be informed to comply with necessary guidelines.

1. **Storage of data/information**

Data storage should meet the requirements of the Research Conduct Policy and the Research Data and Primary Materials Management Procedure which can be found in the [Deakin Legislation and Policy Library](http://www.deakin.edu.au/about-deakin/leadership-and-governance/legislation-and-policy-library)<http://theguide.deakin.edu.au/TheGuide/TheGuide2011.nsf/Web?OpenFrameSet&Doc=eebaea22b17b2d45ca257880001b3fc7?OpenDocument>. In most cases data should be stored securely at Deakin, for a period of at least five years after the final publication of the research outcomes. If the data will be stored in another location, please explain this, and how data security will be maintained. You should include:

- whether the data will be identified/re-identifiable/non-identifiable
- how security will be maintained (locked storage, secure server, etc.)
- how long the data will be stored and
- if and when the data will be disposed of and how security will be maintained.

(See Section 10.8 of the Deakin Guidelines for more information.)

Data will be deleted from Qualtrics when it is downloaded and stored in a password protected file on the Deakin Server available to the research team only and stored at least 5 years from the date of final publication of results. Data may be shared with other researchers and re-used for further research relating to food retail research, beyond the current research project specified.

1. **Collaborative research**

If the research involves multiple researchers collaborating on collection, storage and/or analysis of data, please outline your arrangements for:

- Custodianship of the data
- Storage, retention and destruction of the data or materials
- Rights of access to the data or information
- Rights to analyse or use and re-use the data or information and
- Rights to produce research outputs based upon the data

Deakin University researchers will own the data collected and will only receive the data in its raw anonymised form. The data will be available to collaborators for re-analysis and reuse of relevant research.

1. **Intellectual property, copyright and ownership**

Please detail any intellectual property (individual, community, organisational, commercial), ownership or copyright arrangements related to the data or outputs of the research:

The IP is retained by the research team listed in this application. No commercial outcomes are anticipated.

## Publication of results

(See [Section 4](http://www.nhmrc.gov.au/guidelines/publications/r39) of the [Australian Code for the Responsible Conduct of Research](https://nhmrc.gov.au/about-us/publications/australian-code-responsible-conduct-research-2018) for more information.)

Whose responsibility will it be to notify participants of the outcome of the research?

The research team will be responsible for notifying participants of outcomes, if participants email the lead researcher with a request for results summary.

How will you notify participants of the outcome of the research?

Upon completion of this research, a summary of results will be produced. A summary of results will be provided to the recruiting company for access by the participants. These summary results may be made publicly available on the Deakin University websites and may be published in a scientific paper, which will be publicly available.  A summary of survey findings will be available by June 2021, on request by contacting [redacted].

How will your research be reported/published?

Research results will be published in a peer reviewed journal. Research findings dissemination may also include lay summaries and infographics to target engagement of consumers, advocacy groups and policy makers. This may include dissemination through the Obesity Policy Coalition networks (by Dr Martin) including directly to policy makers to inform current policy consultations. As well as this, Prof Ni Mhurchu (from the University of Auckland) and other members of the research team have close ties to policy makers through previous projects and committee memberships.

How will you manage participant confidentiality?

No identifiable participant data will be collected for the online study and the research team will not have access to such information.

**PART D: Declarations**

**1** I/We, the undersigned declare that the information supplied in this application is true and accurate to the best of my/our knowledge.

I / We the undersigned have read the *National Statement on Ethical Conduct in Human Research* and accept responsibility for the conduct of the project detailed in this application in accordance with the principles contained in the Statement and any other conditions laid down by Deakin University or the Human Ethics Advisory Group.

I/We the undersigned, declare that where the research project may involve contact with a child or young person under the age of 18, I/we have a current Working with Children Check.

Where the project involves a student researcher, as the supervisor I accept responsibility for ensuring that ethics approval is obtained prior to commencing the research and for overseeing the ethical conduct of the project as detailed in the ethics application.

## Signatures: *[redacted]*

**2 ACKNOWLEDGEMENT OF HEAD OF SCHOOL*/DIRECTOR OF RESEARCH OR THEIR NOMINEE**

I the undersigned acknowledge that the Faculty has considered and approved the academic worth of the project described in this application.

Name:

Title:

Signature:       Date:

*If the Head of School (or similar) is also a member of the research or supervisory team, a more senior member of University staff e.g. Dean or Associate Dean (Research) must sign the project as authorising officer.

**Part E: Attachments**

Have you attached the following?

**Yes No  N/A** A copy of the email or certificate confirming successful completion of the online human ethics quiz (first time applicants) or project id of an ethics application/s on which you are listed. For more information on the quiz, copy and paste the following link into your browser: <http://www.deakin.edu.au/students/research/research-support-and-scholarships/integrity-secure/human-ethics/human-research-ethics-training>

**Yes No  N/A** A copy of any advertisements/flyers or other recruitment materials. All advertisements (both written and spoken) must include the following statement: “This study has received Deakin University ethics approval (reference number: insert reference number here).”

**Yes No  N/A** A copy of the Plain Language Statement and Consent Form (PLSCF) or other consent materials to be used in the project

**Yes No  N/A** A copy of any survey, list of questions/topics for interviews, or other materials to be used in this project. Please note Deakin University’s preferred online survey platform is Qualtrics. More information on Qualtrics can be found [here](https://researchsurveys.deakin.edu.au/ControlPanel/) or for technical assistance contact [eresearch@deakin.edu.au](mailto:eresearch@deakin.edu.au)

**Yes No  N/A** Any other documents to be supplied to the participants or used in the conduct of the project

**Yes No  N/A** A letter of support from the organisation/s involved or an organisational PLSC if you are proposing to recruit participants through an external organisation/s

**Yes No  N/A** A completed Organisational Consent Form Coversheet (available on the [Application Forms page](https://www.deakin.edu.au/students/research/research-support-and-scholarships/integrity-secure/human-ethics/human-ethics-forms-and-guidelines/application-forms)) if you are recruiting Deakin staff and students

**Please submit all documents via email to your faculty’s HEAG:**

Faculty of Arts and Education [artsed-ethics@deakin.edu.au](mailto:artsed-ethics@deakin.edu.au)

Faculty of Business and Law [blethics@deakin.edu.au](mailto:blethics@deakin.edu.au)

Faculty of Health [health-ethics@deakin.edu.au](mailto:health-ethics@deakin.edu.au)

Faculty of Science, Engineering and [sciethic@deakin.edu.au](mailto:sciethic@deakin.edu.au)

Built Environment

| Please note: if the hyperlinks in this form result in an error message, return to the form and:   1. *right click on the hyperlink* 2. *click on Edit Hyperlink* 3. *copy the URL to your browser.* |
| --- |

Deakin University is collecting your personal information on this form for the primary purpose of processing your human research ethics application. It will also use this information for monitoring your compliance with the approved protocol. For these purposes Deakin may also provide this information to potential research participants, past or current research participants, or other interested parties in your research. You are not required to provide the information requested, however if the information is not provided, Deakin may not be able to process your ethics application. Deakin manages personal information it holds, including requests by individuals for access to their personal information, in accordance with the Privacy and Data Protection Act 2014 (Vic). Deakin’s Privacy Policy may be viewed on Deakin’s [Policy Library](https://policy.deakin.edu.au/?_ga=1.41072994.1915361819.1415758364). Information on privacy at Deakin is available at <http://www.deakin.edu.au/footer/privacy>.  Questions about privacy may be directed to the Privacy Officer on (03) 5227 8524 or by email to [privacy@deakin.edu.au](mailto:privacy@deakin.edu.au).

**Part F: Glossary**

**Health Data:**

(a) information or an opinion about:

i. the physical, mental or psychological health or a disability (at any time) of an individual; or

ii. an individual’s expressed wishes about the future provision of health, disability or aged care services to him or her; or

iii. a health, disability or aged care service provided, or to be provided, to an individual; that is also personal information; or

(b) other personal information collected to provide, or in providing, a health, disability or aged care service; or

(c) other personal information about an individual collected in connection with the donation, or intended donation, by the individual of his or her body parts, organs or body substances; or

(d) personal information that is genetic information about an individual in a form which is or could be predictive of the health (at any time) of the individual or any of his or her descendants.

**Sensitive Data:**

means information or an opinion about an individual’s:

- racial or ethnic origin; or
- political opinions; or
- membership of a political association; or
- religious beliefs or affiliations; or
- philosophical beliefs; or
- membership of a professional or trade association; or
- membership of a trade union; or
- sexual preferences or practices; or
- criminal record; or
- health information about an individual, or
- genetic information about an individual that is not otherwise health information; or
- biometric information that is to be used for the purpose of automated biometric verification or biometric identification; or biometric templates.

**Reference list:**

1. Mela D, Woolner E. Perspective: total, added, or free? What kind of sugars should we be talking about? Advances in Nutrition. 2018;9(2):63-9.

2. World Health Organization.” Sugar intake for adults and children: guideline”. Geneva, Switzerland: WHO; 2015.

3. Backholer K, Mannan HR, Magliano DJ, Walls HL, Stevenson C, Beauchamp A, et al. Projected socioeconomic disparities in the prevalence of obesity among Australian adults. Aust N Z J Public Health. 2012 Dec;36(6):557-63.

4. Billich N, George NA, Gupta A, Blake MR, Huse O, Backholer K, Boelsen-Robinson T, Peeters A. “The effect of sugar-sweetened beverage labels and signage on consumer knowledge, attitudes and behaviour: A rapid review” [manuscript under review].

5. Billich N, Blake MR, Backholer K, Cobcroft M, Li V, Peeters AJA. The effect of sugar-sweetened beverage front-of-pack labels on drink selection, health knowledge and awareness: An online randomised controlled trial. 2018;128:233-41.

##### **Appendix 1: Online choice survey**

**Screening:**

1. **What is your age?**

- 18 to 25 years
- 26 to 35 years
- 36 to 45 years
- 46 to 55 years
- 56 or over

1. **Are you an Australian resident?**

- Yes
- No

1. **Are you the main person responsible for buying groceries in your household?**

- Yes- I have sole responsibility
- Yes- I share this responsibility
- No- someone else is responsible

1. **How often would you go to the supermarket or complete an online supermarket order for your household?**

- Twice a week or more
- Less than twice a week but more than once per month
- Once a month or less frequently

1. **Which of the following products have you purchased in person or online from a supermarket in the last month for you or your household? (select all that apply**)

- Yoghurt or custard
- Non-alcoholic packaged beverages excluding milk and milk alternatives
- Breakfast cereals
- None of the above

1. **Which of the following do you purchase at least one month or more for your household from the following options?**

*Please select all that are relevant*

- Flavoured yoghurts
- Custard
- Non-diet soft drinks
- Non-diet iced tea
- Fruit drinks
- Flavoured waters
- Chocolate-flavoured cereals
- Toasted muesli or granola
- Kids’ cereal (e.g. Cheerios, Fruit loops)
- Plain yogurt
- Flavored Greek yoghurt
- Plain Greek yoghurt

1. **Do you usually shop for other adults or children?**

- Yes
- No

*If yes*

1. **Please indicate how many adults and how many people you usually shop for?**

- __Adults (18 years or older)
- __Children (less than 18 years)

# Choice Set Questions Introduction (control)

Please read the information below carefully:

For this survey, imagine that you are conducting your usual shopping trip for your household at the supermarket (either online or in person). On your shopping list you intend to purchase a packaged non-alcoholic drink, a yoghurt, and a breakfast cereal.

Below you will be given different branded products to choose from. Select the option you would buy in this situation or choose 'no item' if you would walk away without buying any item in that food category.

1. For the following question imagine you have gone into a supermarket with the intention to buy a pre-packaged non-alcoholic drink. Click on the drink you would buy.

[10 branded product images removed for copy right reasons]

- No drink

*If the participants select no drink*

1a. You selected that you would leave the supermarket without having purchased a product in that product category even though it was on your shopping list. Is this correct?

- Yes
- No

Is there another specific product would you have selected if that option was available? Please specify the product and brand.

_________________________

# Choice Set Questions (control)

1. For the following question imagine you have gone into a supermarket with the intention to buy a yoghurt. Click on the option you would buy.

[10 branded product images removed for copy right reasons]

- No Yoghurt

*If the participants select no yoghurt*

1a. You selected that you would leave the supermarket without having purchased a product in that product category even though it was on your shopping list. Is this correct?

- Yes
- No

Is there another specific product would you have selected if that option was available? Please specify the product and brand.

_________________________

# Choice Set Questions (control)

1. For the following question imagine you have gone into a supermarket with the intention to buy a Breakfast cereal. Click on the option you would buy.

*[10 branded product images removed for copy right reasons]*

- No Breakfast cereal

*If the participants select no breakfast cereal*

1a. You selected that you would leave the supermarket without having purchased a product in that product category even though it was on your shopping list. Is this correct?

- Yes
- No

Is there another specific product would you have selected if that option was available? Please specify the product and brand.

_________________________

# Choice Set Questions Introduction (Chile style: advisory labels for foods high in added sugars)

Please read the information below carefully:

For this survey, imagine that you are conducting your usual shopping trip for your household at the supermarket (either online or in person). On your shopping list you intend to purchase a packaged non-alcoholic drink, a yoghurt, and a breakfast cereal.

Below you will be given different branded products to choose from. Select the option you would buy in this situation or choose 'no item' if you would walk away without buying any item in that food category.

1. For the following question imagine you have gone into a supermarket with the intention to buy a pre-packaged non-alcoholic drink. Click on the drink you would buy.

*[10 branded product images removed for copy right reasons]*

- No drink

*If the participants select no drink*

1a. You selected that you would leave the supermarket without having purchased a product in that product category even though it was on your shopping list. Is this correct?

- Yes
- No

Is there another specific product would you have selected if that option was available? Please specify the product and brand.

_________________________

# Choice Set Questions (Chile style: advisory labels for foods high in added sugars)

1. For the following question imagine you have gone into a supermarket with the intention to buy a yoghurt. Click on the option you would buy.

*[10 branded product images removed for copy right reasons]*

- No yoghurt

*If the participants select no yoghurt*

1a. You selected that you would leave the supermarket without having purchased a product in that product category even though it was on your shopping list. Is this correct?

- Yes
- No

Is there another specific product would you have selected if that option was available? Please specify the product and brand.

_________________________

# Choice Set Questions (Chile style: advisory labels for foods high in added sugars)

1. For the following question imagine you have gone into a supermarket with the intention to buy a Breakfast cereal. Click on the option you would buy.

*[10 branded product images removed for copy right reasons]*

- No Breakfast cereal

*If the participants select no breakfast cereal*

1a. You selected that you would leave the supermarket without having purchased a product in that product category even though it was on your shopping list. Is this correct?

- Yes
- No

Is there another specific product would you have selected if that option was available? Please specify the product and brand.

_________________________

**Post choice experiment questions:**

1. **Thinking about the choices you just made, which of the following did you consider in making your decision?**

(select all that apply)

- Taste
- Familiarity with product
- Healthiness of the item
- Family preferences
- Time of day
- Other _____

1. **Please select all the following statements you agree with**

*[branded image of popular high sugar softdrink]*

**Drinking the above item would…**

(select all that apply)

- …lead to weight gain
- …increase my risk of heart disease
- …have no impact on my health
- …increase my risk of diabetes
- …help me live a healthier life
- …increase my risk of dental decay
- …increase my productivity
- …make social occasions more enjoyable

1. **Please select all the following statements you agree with**

*[branded image of oat-based cereal]*

**Eating the above item would..**

(select all that apply)

- …lead to weight gain
- …increase my risk of heart disease
- …have no impact on my health
- …increase my risk of diabetes
- …help me live a healthier life
- …increase my risk of dental decay
- …increase my productivity
- …make social occasions more enjoyable

1. **Please select all the following statements you agree with**

*[branded image of chocolate custard]*

**Eating the above item would..**

(select all that apply)

- …lead to weight gain
- …increase my risk of heart disease
- …have no impact on my health
- …increase my risk of diabetes
- …help me live a healthier life
- …increase my risk of dental decay
- …increase my productivity
- …make social occasions more enjoyable

**Post choice experiment questions:**

If high sugar products carried this health message (which provided the number of teaspoons of added sugar in product) on the front of package, what would be your most likely response?

*(select all that apply)*

**This product contains**

**16 teaspoons of added suga**r


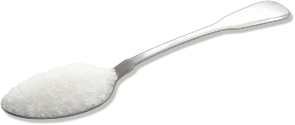


- Buy a smaller package size of the product (if available)
- Buy the product less frequently
- Find a lower sugar alternative
- Stop purchasing this item
- No change in purchasing
- Unsure what I would do
- Other (please specify)____

**Post choice experiment questions:**

If high sugar products carried this health symbol (indicated the healthiness of the item) on the front of package, what would be your most likely response?

*(select all that apply)*


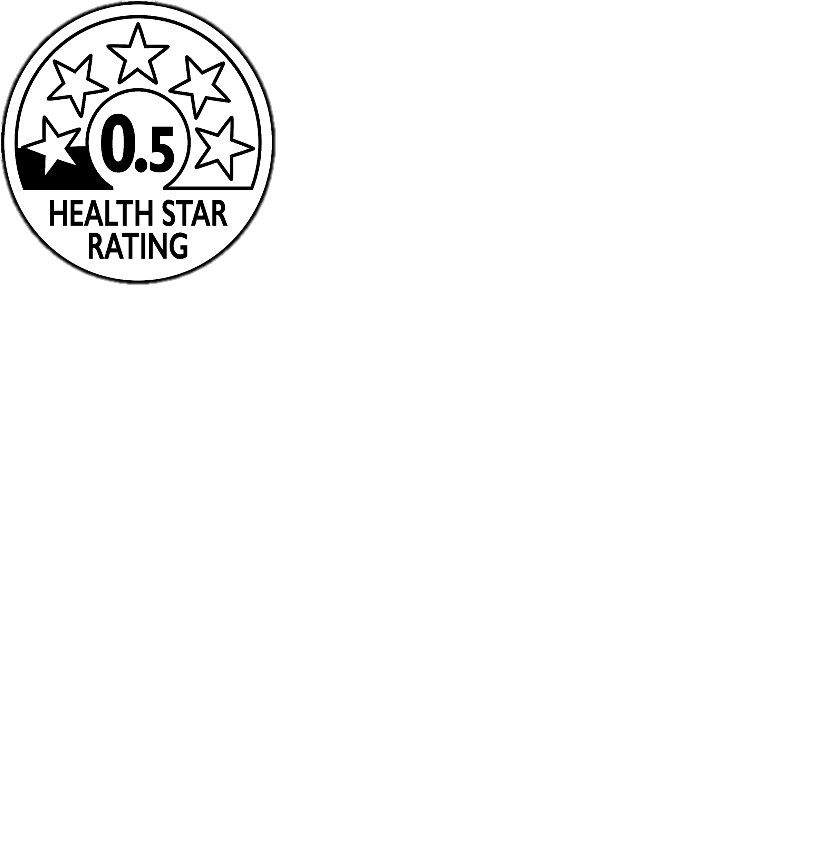
*[Health Star Rating trademarks are owned by the Commonwealth of Australia. Further information on the Health Star Rating can be found at www.healthstarrating.gov.au]*

- Buy a smaller package size of the product (if available)
- Buy the product less frequently
- Find a lower sugar alternative
- Stop purchasing this item
- No change in purchasing
- Unsure what I would do
- Other (please specify)____

**Post choice experiment questions:**

If high sugar products carried this advisory warning on the front of package, what would be your most likely response?

*(select all that apply)*

**HIGH IN SUGAR**

- Buy a smaller package size of the product (if available)
- Buy the product less frequently
- Find a lower sugar alternative
- Stop purchasing this item
- No change in purchasing
- Unsure what I would do
- Other (please specify)____

**Post choice experiment questions:**

If products highlighted which ingredients contained added sugar on the ingredients list on the back of the package, what would be your most likely response?

*(select all that apply)*

Ingredient list: Carbonated water, high fructose corn syrup*, citrate acid, natural flavours, sodium citrate, sodium benzoate

- Buy a smaller package size of the product (if available)
- Buy the product less frequently
- Find a lower sugar alternative
- Stop purchasing this item
- No change in purchasing
- Unsure what I would do
- Other (please specify)____

**Post choice experiment questions:**

If products highlighted added sugar in the nutrition information panel on the back of the package, what would be your most likely response?

*(select all that apply)*

| **NUTRITION INFORMAITON** |  | |
| --- | --- | --- |
| **Servings per package: 8**  **Serving size: 250ml** | | |
| **Av. Quantity** | **Per Serving** | **Per 100ml** |
| **Energy** | **450KJ** | **180KJ** |
| **Protein** | **0.1g** | **0.05g** |
| **Fat total** | **0g** | **0g** |
| - **Saturated** | **0g** | **0g** |
| **Carbohydrates** | **26.5g** | **10.6g** |
| - **Total sugars** | **26.5g** | **10.6g** |
| - **Added sugars** | **26.5g** | **10.6g** |
| **Dietary Fibre** | **0g** | **0g** |
| **Sodium** | **25mg** | **0.5mg** |

- Buy a smaller package size of the product (if available)
- Buy the product less frequently
- Find a lower sugar alternative
- Stop purchasing this item
- No change in purchasing
- Unsure what I would do
- Other (please specify) ___________

**Indicate how strongly you agree with the following statements**

|  | Strongly agree | Agree | Neither agree nor disagree | Disagree | Strongly disagree |
| --- | --- | --- | --- | --- | --- |
| Government should require higher standards to ensure that food corporations clearly identify high sugar levels in products. |  |  |  |  |  |
| Consumers  need more information to make informed decisions about healthy food products. |  |  |  |  |  |
| We need to set higher standards for how the food industry labels the food we eat. |  |  |  |  |  |

**Demographics:**

1. **What is your gender?**

- Women
- Man
- Non-binary/ gender diverse
- My gender identity is not listed
- Prefer not to say

1. **What is your post code?**

**_______**

1. **Is English in your first language?**

- Yes
- No

1. **Are you of Aboriginal or Torres Strait Islander origin?**

- No
- Yes, Aboriginal
- Yes, Torres Strait Islander
- Yes, both Aboriginal and Torres Strait Islander

1. **What is the highest level of education you have completed?**

- Primary school or below
- High school year 11 or below
- Completed high school (year 12, form 6 HSC)
- TAFE or Trade Certificate or Diploma
- Undergraduate university degree (i.e., bachelor, diploma)
- Postgraduate university degree (i.e., postgraduate diploma, masters, PhD)

1. **Before tax is taken out, which of the following ranges best describes your household’s income over the last 12 months? Please include income from all sources, including wages, investments and government pensions and benefits.**

- <=24,999
- 25,000 to 38,999
- 39,000 to 52,999
- 53,000 to 69,999
- 70,000 to 88,999
- 89,000 to 109,999
- 110,000 to 134,999
- 135,000 to 168,999
- 169,000 to 222,999
- >=223,000

1. **Which of these best describes your current employment status?**

- Employed full-time
- Employed part-time
- Self-employed/ freelancer
- Unemployed
- Unable to work due to COVID-19
- Retired
- Not employed – a student
- Not employed – engaged in home duties
- Not employed – unable to work
- Other (please specify)____

1. **Do you know your current height (without shoes)? (if you select yes you will be asked to provide your height)**

- Yes
- No
- I would prefer not to answer

If yes:

1. **Please enter the parameters you would like to enter your height in.**

- centimetres
- feet-inches

1. **How tall are you (without shoes)? You do not need to provide units.**

- centimetres ________________

**21. How tall are you (without shoes)? You do not need to provide units.**

- feet-inches _______ - ________

1. **Do you know your current weight in light clothing? (if you select yes you will be asked to provide your height)**

- Yes
- No
- I would prefer not to answer

If yes:

1. **Please enter the parameters you would like to enter your weight in.**

- kilograms
- stone-pounds

1. **What is your current weight in light clothing?**

- kilograms _________________

1. **What is your current weight in light clothing?**

- stone - pounds ______ - ______

1. **What best describes your current goals for your weight?**

- I am trying to lose weight
- I am trying to gain weight
- I am trying to maintain my weight (neither gain nor lose)
- I do not have a goal for my weight

1. **In the past week, on how many days have you done a total of 30 minutes or more of physical activity, which was enough to raise your breathing rate? This may include organized sport, exercise, or brisk walking or cycling for recreation or to get to and from places but should not include housework or physical activity that may be part of your job. Provide an answer between 0 and 7 days.**

- ______ days

1. **When you complete your supermarket shop, who are you usually buying food items for?**

Select all that apply

- Myself
- One or more children younger than 18 years in my household
- Another adult over the age of 18 years in my household

1. **Which of these is the main source of food you purchase for your household?**

- Convenience store
- Supermarket
- Greengrocer or other specialty store
- Fast food/restaurant/café
- Online
- Meal kit delivery

**Thank you for your time and assisting us with this survey. When you click 'Submit' the survey will be complete. If you would like to make any further comments, please enter them below.**

##### **Appendix 2: Plan language statement**

#####

**Plain Language Statement: Supermarket shopping study**

**Date: XXXXXXX**

**Full Project Title:** Exploring effectiveness of policy options for added sugar labelling

**Principal Researcher:** [redacted]

**Associate Researchers:** [redacted]

**Reference Number:** HEAG-H XXXX

**Overview**

You are invited to take part in this online survey to see how providing information about food affects food choices. You have been invited as you have previously agreed to be contacted by a survey recruitment company. You are eligible to participate in this research if you are 18 years or older, are living in Australia, are responsible for completing the grocery shopping in your household, are a regular (at least once a month or more) purchasers of breakfast cereal, yoghurt and non- alcoholic pre-packaged drinks for yourself or household and live with at least one child (less than 18 years).

This Plain Language Statement contains detailed information about the research project. Please read this Statement carefully. You should print off a copy of the Statement to keep as a record.

If you agree to participate in this study, you will be asked to complete a survey of approximately 10 minutes asking about your food preferences from a range of options, as well as several demographic questions. This research may assist in understanding how providing information about food items can influence purchasing behaviours.  We do not foresee any potential risks to participants.

Participants will be reimbursed directly by the recruiting company. Please contact them directly for details of reimbursement.

**Privacy, Confidentiality, and Disclosure of Information**

Data collected for this study will not be associated with any information that identifies you. All data will be stored electronically in a secure drive within the School of Health and Social Development at Deakin University. Data will be stored securely for five years after publication, after which all data will be destroyed. In any publication or report all information will be anonymous.  Data may be shared with other researchers and re-used for further research relating to food retail research, beyond the current research project specified.

**Results of the Project**

Upon completion of this research, a summary of results will be produced. These summary results may be made publicly available on the Deakin University website and may be published in a scientific paper, which will be publicly available.  A summary of survey findings will be available by June 2021, on request by contacting [redacted].

**Participation is Voluntary**

Participation in this survey is voluntary and participants have the right to withdraw at any stage by closing the survey. This is an anonymous survey; your information once submitted cannot be identified or withdrawn. If you agree to take part in this research, please complete the survey. The completion of the survey will be taken as agreement for your information to be used in this project.

**Funding and ethical oversight**

This project is funded by a Deakin University Faculty of Health HAtCH grant ($28,888). Approval to undertake this research project has been given by the Human Research Ethics Committee of Deakin University.

**Complaints**

If you have any complaints about any aspect of the project, the way it is being conducted or any questions about your rights as a research participant, then you may contact:

The Human Research Ethics Office, Deakin University, 221 Burwood Highway, Burwood Victoria 3125, Telephone: 9251 7129, XXX@deakin.edu.au

Please quote project number [201X-XXX].

**Contact**

If you require more information or if you have any problems concerning this project, you can contact either of the researchers responsible for this project:

*[Contact details of researchers]*

- **I have read the plain language statement and I agree to participate *(Clickable)***
- **I do not agree to participate *(Clickable)***
