# Supplementary material for: Exploring the effects of added sugar labels on food purchasing behaviour in Australian parents: An online randomised controlled trial
Source: PLoS One. 2022 Aug 25;17(8):e0271435. doi: 10.1371/journal.pone.0271435 (PMC9409597; doi:10.1371/journal.pone.0271435)
Supplement: S2 File — (DOCX) [file pone.0271435.s002.docx]

**Exploring effectiveness of policy options for added sugar labelling**

Internal protocol April 2020 v 2.1

## Trial name for publication

- TBD

## Trial name for participants (options)

- Food Shopping Decisions Study

## Principal investigator:

Miranda Blake

## Associate investigators:

Devorah Riesenberg

Anna Peeters

Kathryn Backholer

Jane Martin

Cliona Ni Mhurchu

## Proposed authors and order of resulting publication:

Devorah Riesenberg, Anna Peeters, Kathryn Backholer, Jane Martin, Cliona Ni Mhurchu, Miranda Blake

# Summary

This internal protocol outlines two online randomised control trials (RCT) to test the relative effects of added sugar labelling on Australians’ intended food and beverage purchases. It will be adapted for the Australia New Zealand Clinical Trials Registry and ethics applications. Australian adults aged 18 years and older will be recruited using a recruitment agency. Trial 1 (n=1,008) will determine the relative impact of 7 different added sugar labelling options on healthiness of intended purchases of breakfast cereals, yoghurts and non-alcoholic packaged beverages. Trial 2 (n=1,152) will examine: (i) the relative effect of different combinations of multi-labelled food and beverage products (informed by the two most effective labels in Trial 1); and (ii) effects by level of participant sugar consumption, gender, age, education, and income. This new evidence will guide and inform the development of effective labelling policies to reduce added sugar consumption for Australians and New Zealanders.

## Background:

“Added” sugars include sugar added to foods and beverages in the production or cooking process (1). “Free” sugars are a broader category of sugars, and include “all monosaccharides and disaccharides added to foods by the manufacturer, cook, or consumer, plus sugars naturally present in honey, syrups, and fruit juices and fruit juice concentrate” except those that are naturally occurring or present in foods such as fruits, vegetables and milk (2). For consistency, in this protocol we will refer to any sugar-based ingredient added to food as “added” sugar and this may include “free” sugar as well, as per the terminology used by the Food Regulation Standing Committee (FRSC) policy consultation paper on sugar labelling (3) which is the prompt for this study. Over half of Australians (52%) exceed the World Health Organization (WHO)’s recommended intake of added sugar (2), increasing their risk of obesity, Type 2 Diabetes, dental decay, and some cancers. Further, the majority (90%) of Australians also exceeded the WHO conditional recommendation that added sugars be reduced to less than 5% of energy intake, where additional healthy benefits have been shown (2). Added sugar intake, and the burden of related chronic diseases, are disproportionately higher for disadvantaged populations in Australia (4). While there is empirical evidence that increased nutrition knowledge increases dietary quality (5, 6), packaged food in Australia is not currently required to display added sugar content, either on front-of-package or in the nutrition information panel on the back or side of the pack. This is an area of strong public and policy interest internationally. There is moderate to strong evidence from laboratory trials that front-of-package added sugar warning labels reduce intended purchasing of sugar-sweetened beverages (7). This includes evidence from our previous online randomised control trial (RCT) which demonstrated that front-of-package labels, including graphic warning labels, reduced intention to purchase sugar-sweetened beverages among Australians aged 18-35 years (8). However, there is limited evidence of labelling effects on actual or intended food purchases for foods other than sugar-sweetened beverages. The Australian and New Zealand Government Food Regulation Standing Committee (FRSC) is exploring options for labelling of added sugars on packaged foods and drinks available for sale in Australia and New Zealand, to support consumers in making informed choices to reduce their added sugar intake. FRSC has identified a number of literature gaps hindering policy development, including evidence: (i) on labelling for products other than sugar-sweetened beverages, including seemingly “healthy” food products such as flavoured yoghurt and breakfast cereal; (ii) involving Australian and New Zealand consumers; and (iii) on the differential impact for different population subgroups (3). While FRSC is considering policies requiring display of multiple sugar labels simultaneously, we are not aware of evidence to date testing the effect of displaying multiple added sugar labels at once.

This new evidence will be critical to the development of effective labelling policies to reduce Australian and New Zealand added sugar consumption.

Research question:

#### Primary

What is the impact of added sugar labels on intended purchase of selected high sugar food and drink products?

#### Secondary

What is the impact of different combinations of added sugar labels on intended purchase of selected high sugar food and drink products?

How does the effect of added sugar labels on intended purchase of selected high sugar food and drink products differ according to participant characteristics (including age, gender, education, income, or usual levels of sugar consumption)?

Hypothesis:

#### Primary

Added sugar labelling on food and drink items reduces the proportion of intended purchases that are high sugar products, compared to no added sugar labels.

#### Secondary

Displaying multiple added sugar labels on food and drink items reduces the proportion of intended purchases that are high sugar products, compared no labels or display of one added sugar label.

Participant characteristics including age, gender, education, income, and usual levels of sugar consumption alter the effect added sugar labelling on intended purchasing.

## Methods:

### Overall approach:

The project will consist of two phased online RCTs. The results from Phase 1 (Trial 1), will inform the design of Phase 2 (Trial 2). Online RCTs are useful for testing policies that are not yet implemented in the real world, and are able to test relative effectiveness across a number of different scenarios.

#### Trial 1 methods:

We will examine the relative effectiveness of different labelling options for three food product groups (breakfast cereals, yoghurts and non-alcoholic packaged beverages). These food groups have been chosen as they make a significant contribution to Australians’ energy intake, and because there is a wide variation in added sugar content of available products. According to the Australian National Health Survey (AHS) 2011-12, overall 36% of Australians and 16% of Australians consumed ready to eat breakfast cereals and yogurt, respectively, in the 24 hours prior to the survey. Additionally, just over half (52%) of added sugars in the diet were consumed from beverages, with the leading beverages being soft drinks, electrolyte and energy drinks (19%), fruit and vegetable juices and drinks (13%), and cordial (4.9%) (4).

1,008 Australian adults aged ≥18 years reporting regular (once a month or more) purchase of yoghurt, breakfast cereals and non-alcoholic packaged beverages will be recruited through an online panel provider. Sample size calculations are detailed below. Eligible participants will be randomised to complete one of seven online survey conditions in Qualtrics. Each survey condition will include of three hypothetical purchasing tasks: one each for breakfast cereals, yoghurts and non-alcoholic packaged beverages. Each task will consist of selecting between ten different products with varying sugar content, labelled with one of seven labelling options (depending on the survey condition) currently used in Australia or internationally or under consideration by the Australian Government: (i) Status quo – current Nutrition Information Panel (NIP) (Back of Pack labelling system without added sugar content) (control condition); (ii) added sugars quantified in the NIP (the remaining five conditions will also include a back-of-pack NIP with added sugar content); (iii) Pictorial approaches to convey the amount or types of sugars in a serving of food (teaspoons of sugar); (iv) Chilean-style advisory labels for foods high in added sugars; (v) Health Star Rating (using current algorithm without added sugar)- simple display without panel; (vi) Health Star Rating (using proposed algorithm with added sugar)- simple display without panel; and (vii) Change to statement of ingredients to asterisk ingredients with added sugar. Advisory labels will be displayed on high sugar products only. All other labels will be displayed on every product in the choice set, as relevant. Each participant will be exposed to one treatment condition (sugar labelling intervention or control) and complete the purchasing task in all three food categories (Figure 1).

| Label 1: Status quo – current Nutrition Information Panel (NIP) (Back of Pack labelling system without added sugar content) (control condition)  Label 2: Added sugars quantified in the NIP  Label 3: Pictorial approaches to convey the amount or types of sugars in a serving of food  Label 4: Chilean-style advisory labels for foods high in added sugars; (v) Health Star Rating  Label 5 and 6: Health Star Rating  Label 7: Change to statement of ingredients to asterisk ingredients with added sugar | 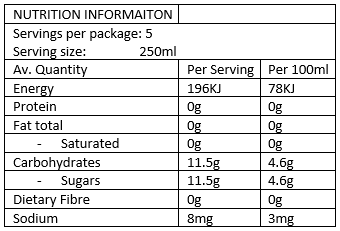  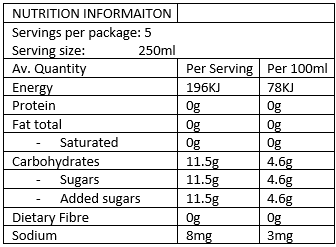  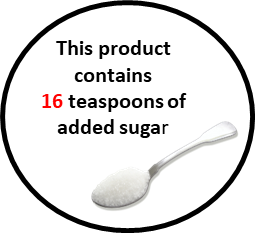  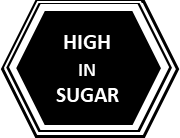  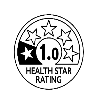  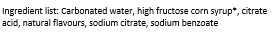 |
| --- | --- |

Figure 1: Examples of intervention labels 1-7 used in the choice experiment study. Health Star Rating trademarks are owned by the Commonwealth of Australia. Further information on the Health Star Rating can be found at www.healthstarrating.gov.au

#### Calculating added sugar content

‘High sugar’ products will be classified using the UK Nutrient Profiling Model (foods >6.25g added sugar/100g and beverages >3.13g/100mL (9).

In order to determine added sugar in branded yoghurt and breakfast cereals we will make use of the AUSNUT database (10) and a standard protocol (11). To calculate added sugar we use the following decision tree: i) if the ingredients list includes added/free sugars only then the total sugars will be considered added sugars; ii) if the ingredients list includes a mixture of added sugars and intrinsic sugars then we will allocate added sugar as a percentage of total sugar content, based on the percentage added sugar content of a similar generic product in AUSNUT database. We will use AUSNUT “free sugars” content to calculate this.

For water-based beverages we will assume that all sugars are added sugars. For fruit juices, we will select branded drinks that provide the percentage of juice the product so we can calculate added sugar content.

To calculate the modified Health Star Rating including added sugar, we will use the UK scale for 10% added sugar intake (12).

#### Selection of food products for testing

Branded food products will be used to increase the realism of choice tasks for participants, in order to maximise external validity. The selection of the 10 food products for testing in each category will be based on:

- Popularity according to Canstar reports (when available)
- Brands available at both Coles and Woolworths supermarkets
- Include a variety of flavours and products sub-categories, for example cereal flakes, cereals with added fruit etc.
- Including products with a range of added sugar contents

#### Piloting

The survey wording and flow will be tested within the research team, and wider research group. Prior to the full Trial 1 recruitment, there will be a ‘soft-launch’ of 100 participants to allow detection and correction of any survey errors. The ‘soft-launch’ will also allow the assessment of whether an appropriate range of branded products have bene included. When a participant has selected to ‘purchase’ no product, they will be asked to specify if there was another product they would have purchased if it was available.

#### Defining high sugar purchasers vs low sugar purchasers

Participants will be asked to select all relevant products they purchase at least once a month for themselves or their household from a range of generic product subcategories in each of the product categories of interest (yoghurt and custards, breakfast cereal and non-alcoholic packaged drinks). For example, in the yoghurt categories this will include plain Greek or natural yoghurt, flavoured Greek or natural yoghurt, custard etc. These generic subcategories will be classified as “high” and “low” sugar products. If the participants selects one or more high sugar products in each category they will be considered a high sugar purchaser. We will implement a quota for half of all participants to be classified as “high sugar purchasers”.

#### Trial 2 methods:

The seven labelling options in Trial 1 are distinct and could feasibly be combined for potential increased effect. We will examine the effect of displaying multiple labels simultaneously and effects for different consumer subgroups. The two most effective labels from Trial 1 will be used to determine four test scenarios for Trial 2: Label A; Label B; Label A+B; Status quo (control condition). 1,152 Trial 2 participants will be recruited.

### Sample size calculations:

Sample sizes of 144 completed participant surveys per subgroup for Trials 1 and 2 are based on detection of 32% difference in intended purchasing of high versus low sugar products, with a statistical significance level of 5% and 80% power (based on Billich et al. 2018 findings, smallest difference detected between baseline purchases [64% purchased sugar sweetened beverages] compared to 28% to 47% participants across treatment groups, and approximately 10% unusable surveys due to errors)(8). Trial 1: 7 treatment groups x 144 participants per group= 1,008 participants. Trial 2: 4 treatment groups x 144 participants per group x 2 for subgroup analysis of high v. low sugar consumers= total 1152 participants.

### Recruitment:

A third-party recruitment company will be used to recruit participants for the online study. The recruitment company have security measures in place to securely store participants’ identifiable data. No identifiable information will be received by the researchers from the recruitment company. Research data (i.e. answers to the survey) will not be available to or stored by the recruitment company.

#### Eligibility

- Australian residents over the age of 18 years
- Access to a computer and internet connection
- Regular (at least once a month or more) purchasers of breakfast cereal, yoghurt and non- alcoholic pre-packaged drinks for themselves or their household
- Completes at least one supermarket shop per month for their household
- Lives with at least one child <18 years at home

### Analysis for Trials 1 and 2:

#### Primary outcome:

The differences in the proportion of high sugar products among intended purchases across treatment groups which will be examined using logistic regression analyses. “High” sugar products will be classified using the UK Nutrient Profiling Model (foods >6.25g added sugar/100g and beverages >3.13g/100ml)(9).

#### Secondary outcomes:

The differences between treatment groups in overall intended purchases by (i) added sugar content (g/100g); (ii) Health Star Rating (from 0.5 to 5); and (iii) percentage of Five Food Group (healthy) and discretionary (unhealthy) foods according to Australian Dietary Guidelines (13), which will be analysed using logistic or linear regression, as appropriate. Trial 2 results will be examined for the whole sample. We will then test for interaction with possible effect modifiers, including gender, age, education, and usual sugar consumption, and stratify analyses when an interaction term is significant at a p-value of <0.01. All analyses will be adjusted for key covariates.

## Ethical approval

A low risk ethics application will be submitted for approval from Deakin University Human Research Ethics Committee. This trial has received funding from the Deakin University Faculty of Health HAtCH 2020 grant, researchers will provide their time in-kind.

## Trial registration

The trial will be registered with Australia New Zealand Clinical Trials Registry (ANZCTR) prior to commencement.

## Data sharing

Deakin University researchers will own the data collected and will only receive the data from the recruitment company in its anonymised form. Anonymous survey data will be available to collaborators for relevant consumer behaviour research.

## Intellectual property considerations

The IP is retained by the research team listed in this application. No commercial outcomes are anticipated.

## Dissemination and publication planning

FSANZ is currently exploring options for added sugar labelling and therefore in order to ensure their policy plan is evidence-based. We will contact FSANZ (supported by EI Martin) and notify them about our project plan and ask for targeted feedback on policy-relevant issues. Further, once results have been finalised but prior to publication we will send them a short research brief to inform them of our results to assist with policy making. We will also send the policy brief to other parties that we believe will be useful for leveraging policy (e.g. Victorian Health Minister and NZ Ministry of Health).

Research results will be published in a peer reviewed scientific journal. Research findings dissemination will also include lay summaries and infographics to target engagement of consumers, advocacy groups and policy makers. This will include dissemination through the Obesity Policy Coalition networks (by EI Martin) including directly to policy makers to inform current policy consultations. As well as this, Cliona Ni Mhurchu (from the University of Auckland) and other research team members have close ties to policy makers through previous projects and committee memberships.

## Timeline

**Figure 1. Timeline for added sugar labelling choice experiment**

Figure 1 demonstrates the expected timeline for the project. It is expected that the project will be completed nine months, with results submitted to a scientific paper December 2020.

## References

1. Mela D, Woolner E. Perspective: total, added, or free? What kind of sugars should we be talking about? Advances in Nutrition. 2018;9(2):63-9.

2. World Health Organization. Sugars intake for adults and children: Guideline. Geneva, Switzerland: WHO; 2015.

3. Food Standards Australia New Zealand. Literature review on consumer knowledge, attitudes and behaviours relating to sugars and food labelling Canberra, Australia: FSANZ; 2017 [cited 2019 23 Jul]. Available from: <http://www.foodstandards.gov.au/>.

4. Australian Bureau of Statistics. Australian Health Survey: consumption of added sugars, Cat no. 4364.0.55.011 Canberra, Australia: ABS; 2016 [updated 27 Apr 2016; cited 2022 10 Jul]. Available from: <http://www.abs.gov.au/>.

5. Kiszko KM, Martinez OD, Abrams C, Elbel B. The influence of calorie labeling on food orders and consumption: a review of the literature. J Community Health. 2014;39(6):1248-69.

6. Spronk I, Kullen C, Burdon C, O'Connor H. Relationship between nutrition knowledge and dietary intake. Br J Nutr. 2014;111(10):1713-26.

7. Gupta A, Billich N, George NA, Blake MR, Huse O, Backholer K, et al. The effect of front-of-package labels or point-of-sale signage on consumer knowledge, attitudes and behavior regarding sugar-sweetened beverages: a systematic review. Nutr Rev. 2021;79(10):1165-81.

8. Billich N, Blake MR, Backholer K, Cobcroft M, Li V, Peeters A. The effect of sugar-sweetened beverage front-of-pack labels on drink selection, health knowledge and awareness: An online randomised controlled trial. Appetite. 2018;128:233-24.

9. Public Health England. Annex A: The 2018 review of the UK Nutrient Profiling Model. London, UK: PHE; March 2018.

10. Food Standards Australia New Zealand. AUSNUT 2011–13–Australian food composition database. Canberra: FSANZ; 2014.

11. Louie JCY, Moshtaghian H, Boylan S, Flood VM, Rangan A, Barclay A, et al. A systematic methodology to estimate added sugar content of foods. 2015;69(2):154-61.

12. The George Institute for Global Health. Submission to the 5 year review of the Health Star Rating System- draft review report Sydney, NSW The George Institute for Global Health; 2019 [cited 2021 Aug 18]. Available from: <https://www.georgeinstitute.org.au/sites/default/files/documents/tgi_submission_to_5_year_review_of_hsr_draft_review_report_march_2019_final.pdf>.

13. National Health and Medical Research Council. Australian Dietary Guidelines. Canberra, Australia: NHMRC; 2013.
