# Supplementary material for: Exploring the effects of added sugar labels on food purchasing behaviour in Australian parents: An online randomised controlled trial
Source: PLoS One. 2022 Aug 25;17(8):e0271435. doi: 10.1371/journal.pone.0271435 (PMC9409597; doi:10.1371/journal.pone.0271435)
Supplement: S4 File — (DOCX) [file pone.0271435.s004.docx]

**S4 File. Example choice task for each food category**

*[***Note that in the instructions and example choice sets following, edited branded photos of products which were used in the study have been replaced with mock-up examples due to copyright considerations.]*

Please read the information below carefully:

For the next few questions, imagine that you are conducting your usual shopping trip for your household at the supermarket (either online or in person). On your shopping list you intend to purchase one packaged non-alcoholic drink, one yoghurt or custard, and one breakfast cereal.

You will be given some different branded products to choose from. Select the option you would choose in this situation or choose 'no item' if you would walk away without purchasing any item in that food category.

___

For the following question imagine you have gone into a supermarket with the intention to buy a bottled drink for yourself or your household. Please select the drink you would choose.

You can also click 'Click here to see more details' to see the packet enlarged and see the Nutrition Information Panel and ingredients list.


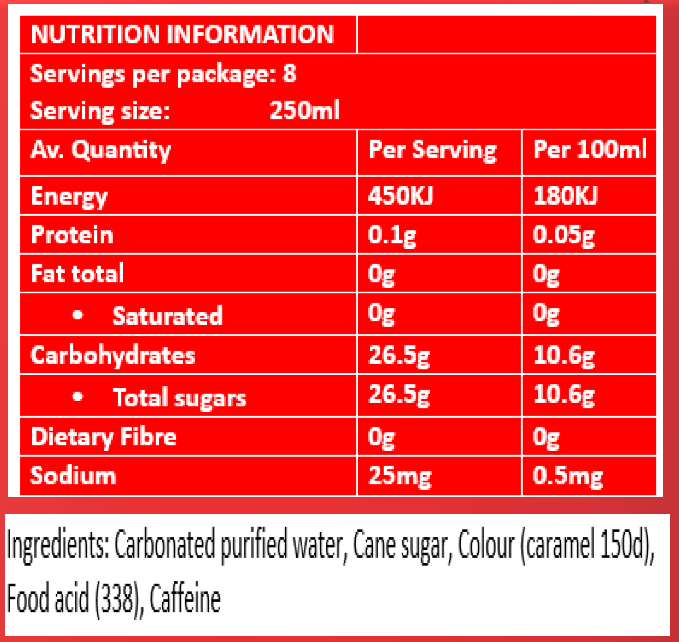

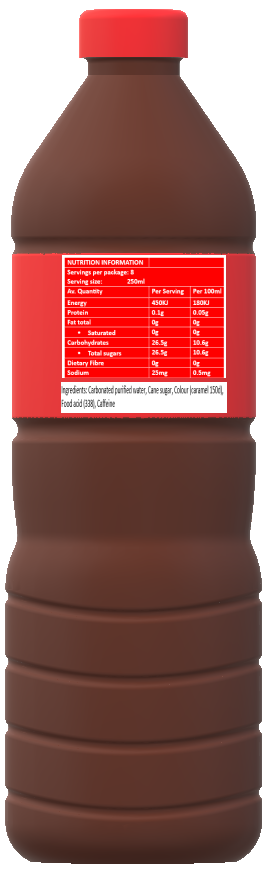

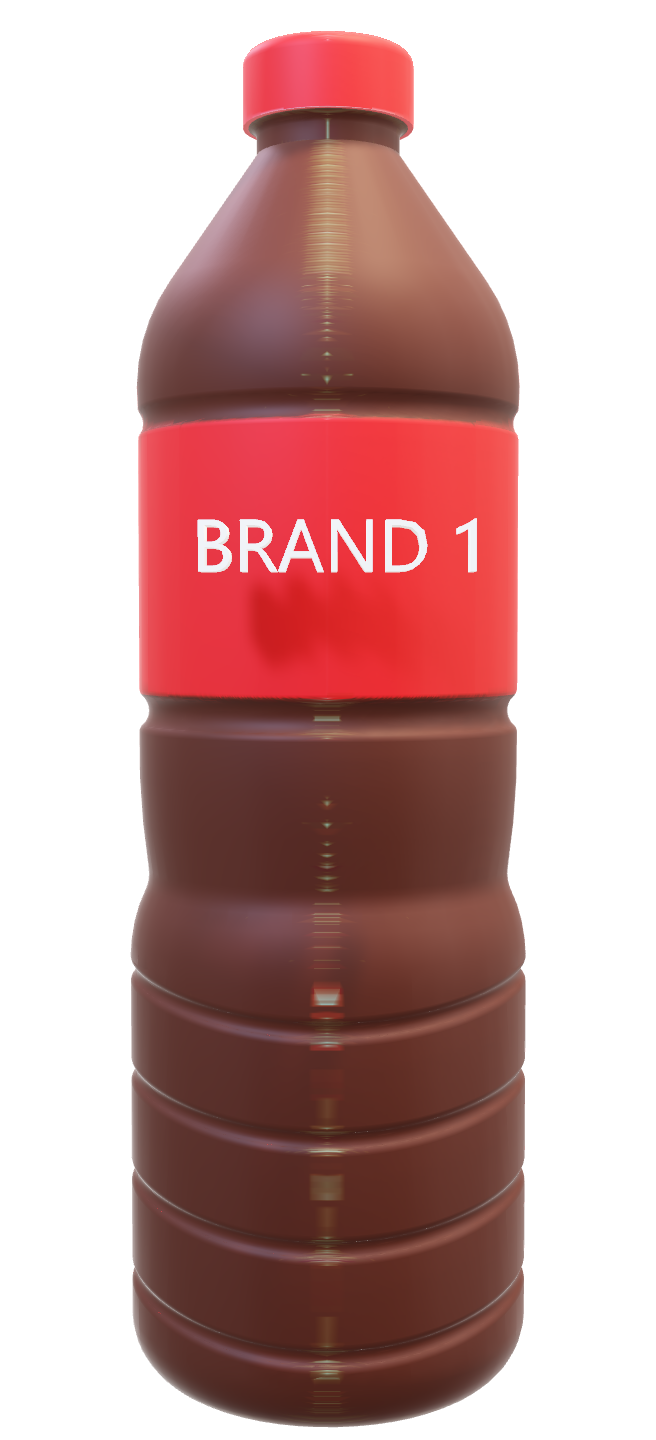


Click to zoom

Click to see back of pack

**Choice Set Questions (Control)**

#
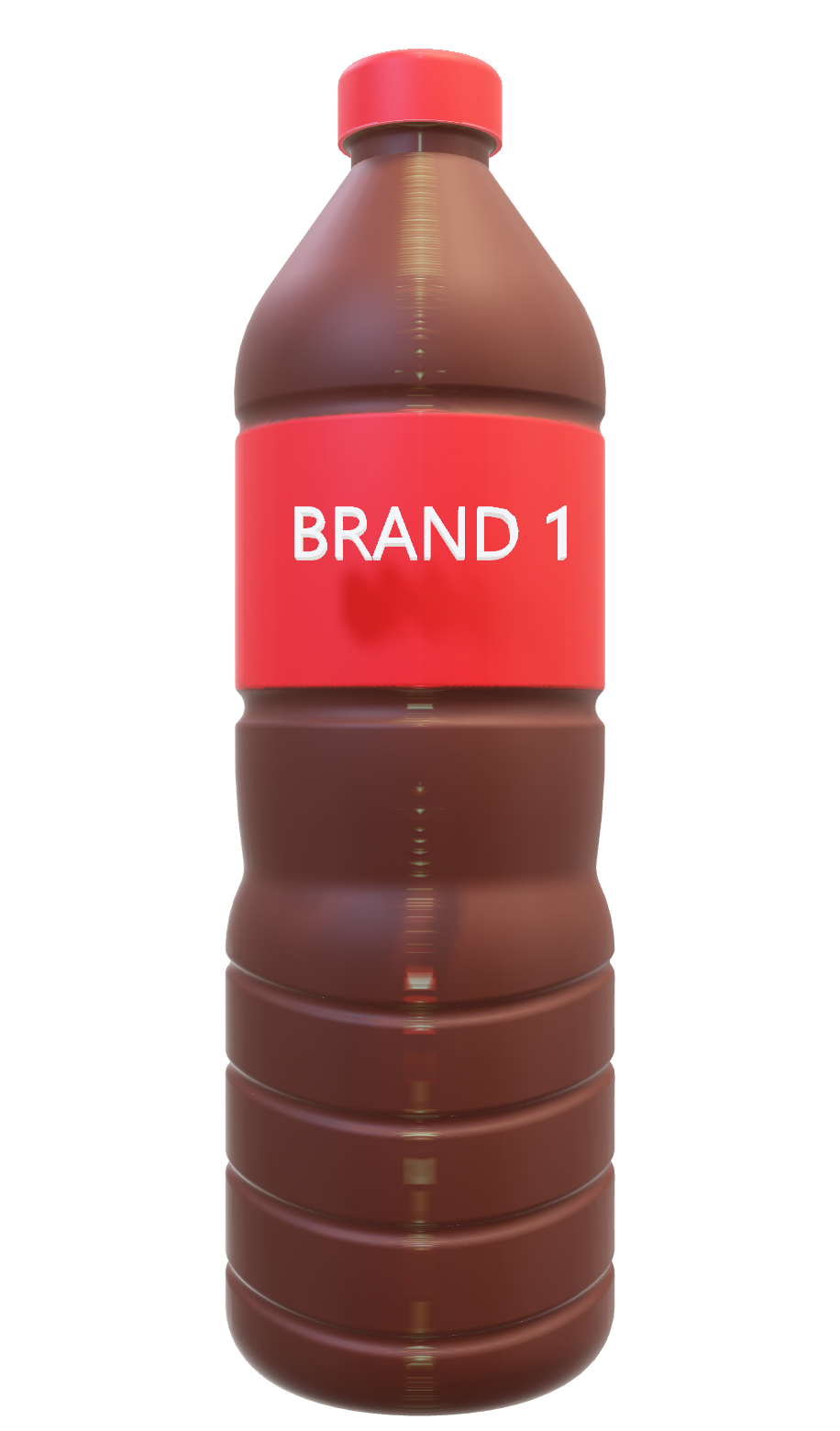
 *[10 full-page branded product photos with unmodified front-of-packs. Photos removed due to copyright considerations.]*

***
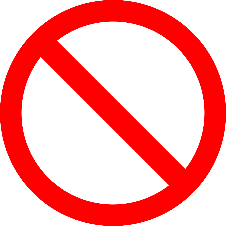
***

No drink

*If the participants select no drink*

1a. You selected that you would leave the supermarket without having purchased a product in that product category even though it was on your shopping list. Is this correct?

- Yes
- No

Is there another specific product would you have selected if that option was available? Please specify the product and brand.

_________________________

**Choice Set Questions (‘Teaspoon of Added Sugar’ label)**

For the following question imagine you have gone into a supermarket with the intention to buy a tub of yoghurt for yourself or your household. Please select the yoghurt you would choose. You can also click 'Click here to see more details' to see the packet enlarged and see the Nutrition Information Panel and ingredients list.


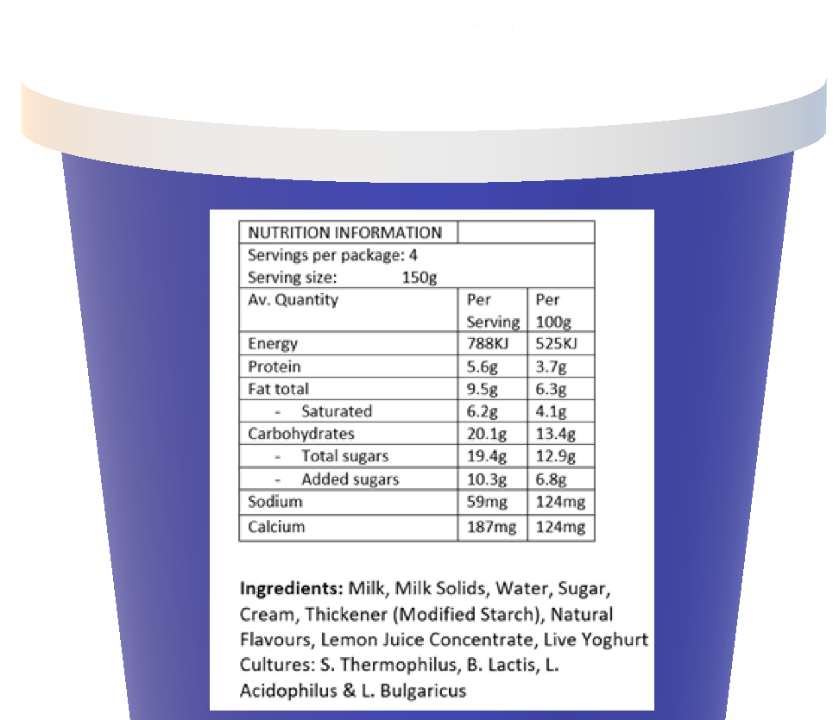

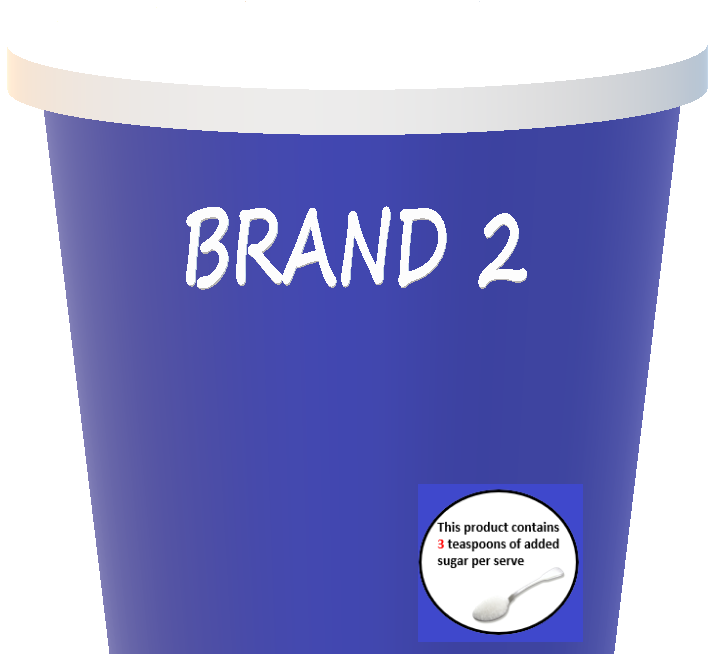

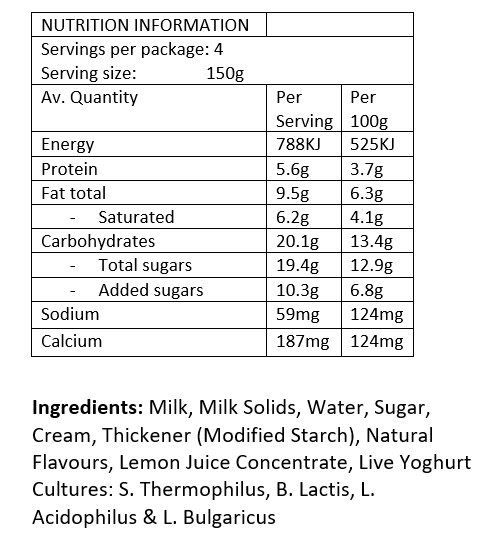


Click to see back of pack

Click to zoom

# *[10 full-page branded product photos with teaspoon of sugar labels on each front-of-pack. Photos removed due to copyright considerations.]*

#
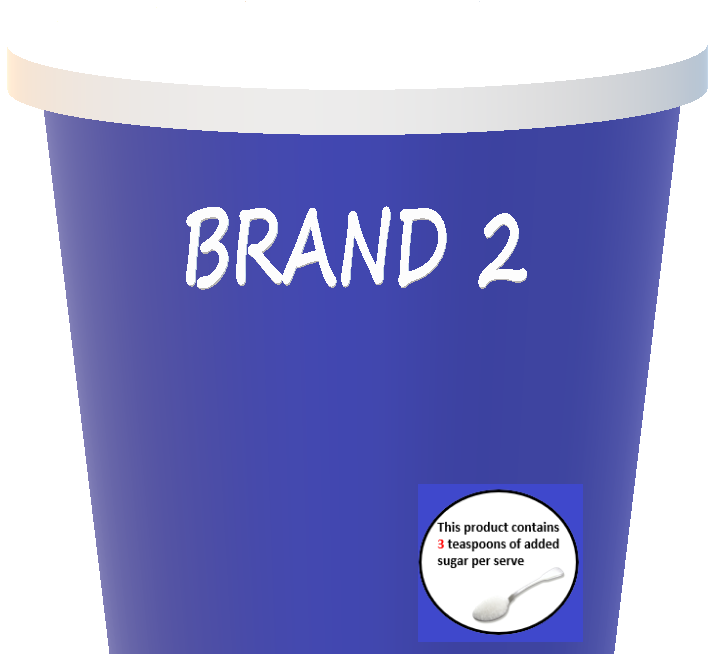


# *
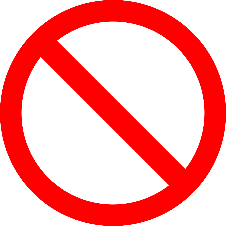
*

No yoghurt

*If the participant selects no yoghurt*

1a. You selected that you would leave the supermarket without having purchased a product in that product category even though it was on your shopping list. Is this correct?

- Yes
- No

Is there another specific product would you have selected if that option was available? Please specify the product and brand.

_________________________

**Choice Set Questions (‘Warning’ label for foods high in added sugars)**

For the following question imagine you have gone into a supermarket with the intention to buy a packet of breakfast cereal for yourself or your household. Please select the breakfast cereal you would choose.

You can also click 'Click here to see more details' to see the packet enlarged and see the Nutrition Information Panel and ingredients list.


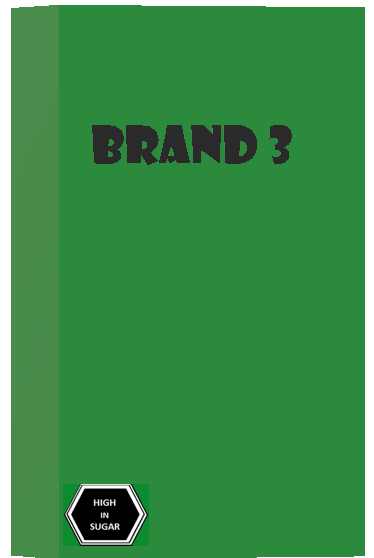

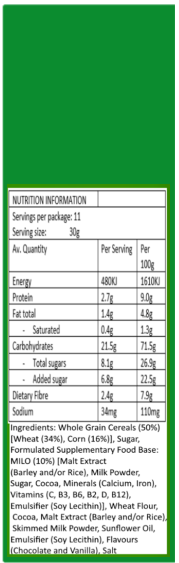

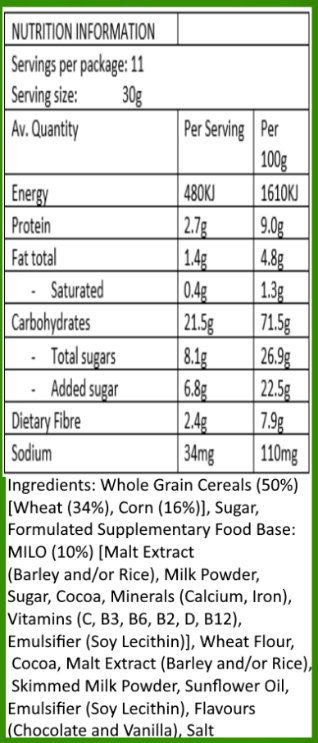


Click to zoom

Click to see back of pack

# *[10 full-page branded product photos with ‘High in Sugar’ warning labels on front-of-pack of high sugar products. Photos removed due to copyright considerations.]*


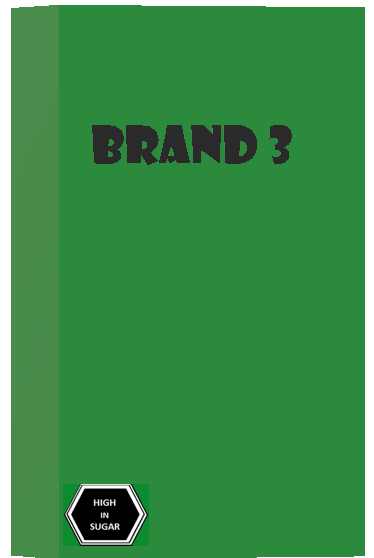


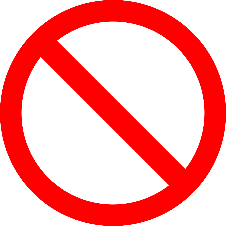


No Breakfast cereal

*If the participant selects no breakfast cereal*

You selected that you would leave the supermarket without having purchased a product in that product category even though it was on your shopping list. Is this correct?

- Yes
- No

Is there another specific product would you have selected if that option was available? Please specify the product and brand.

_________________________
