# Supplementary material for: Exploring the effects of added sugar labels on food purchasing behaviour in Australian parents: An online randomised controlled trial
Source: PLoS One. 2022 Aug 25;17(8):e0271435. doi: 10.1371/journal.pone.0271435 (PMC9409597; doi:10.1371/journal.pone.0271435)
Supplement: S5 File — (DOCX) [file pone.0271435.s005.docx]

**S5 File: Included food product options**

**Table A: Descriptions of non-alcoholic pre-packaged beverages included in choice tasks**

| **Brand** | **Beverage item** | **Item description** | **High/ low sugar classification** | **Package size (mL)** | **Serving size (mL)** | **Per 100g** | | | | **Per serving** | | **HSR using total sugars algorithm** | **HSR using added sugars algorithm** |
| --- | --- | --- | --- | --- | --- | --- | --- | --- | --- | --- | --- | --- | --- |
|  |  |  |  |  |  | **Energy (kJ)** | **Fat (g)** | **Total sugar (g)** | **Added sugars (g)** ^a^ | **Added sugar (g)** ^a^ | **Added sugar (nearest teaspoons)** ^a^ |  |  |
| Coca-Cola | Coca cola (regular) | Soft drink (regular) | High | 2000 | 250 | 180 | 0 | 10.6 | 10.6 | 26.5 | 6 | 0.5 | 0.5 |
| Coca-Cola | Sprite (regular) | Soft drink (regular) | High | 2000 | 250 | 151 | 0 | 8.6 | 8.6 | 21.5 | 5 | 2.0 | 1.5 |
| Golden Circle | Orange fruit drink (25% reconstituted fruit juice) | Juice (less than 99%) | High | 1000 | 200 | 185 | 0 | 9.9 | 9.9 | 19.8 | 5 | 0.5 | 0.5 |
| Lipton | Iced tea (lemon) | Iced tea | High | 1500 | 250 | 93 | <0.1 | 5.2 | 5.2 | 13.0 | 3 | 1.5 | 1.5 |
| Lipton | Iced tea (light peach) | Iced tea (diet) | Low | 1500 | 250 | 5 | <0.1 | 0.1 | 0.1 | 0.3 | 0 | 3.5 | 3.5 |
| Coca-Cola | Coke no sugar | Soft drink (diet) | Low | 2000 | 250 | 1.4 | 0 | 0.0 | 0.0 | 0.0 | 0 | 3.5 | 3.5 |
| Coca-Cola | Sprite no sugar | Soft drink (diet) | Low | 2000 | 250 | 4.2 | 0 | 0.0 | 0.0 | 0.0 | 0 | 3.5 | 3.5 |
| Nudie ^b^ | 100% orange juice | Juice (99% fruit) | High | 2000 | 250 | 180 | 0 | 7.6 | 0.0 | 19.0 | 5 | 3.0 | 4.0 |
| Mount Franklin | Natural lemon flavoured lightly sparkling water | Flavoured sparkling water (non-sugar) | Low | 1250 | 250 | 1.9 | 0 | 0 | 0 | 0 | 0 | 4.5 | 4.5 |
| Mount Franklin | Lightly sparkling water | Sparkling water | Low | 1250 | 250 | 0 | 0 | 0 | 0 | 0 | 0 | 5.0 | 5.0 |

HSR, Health Star Rating

^a^ ‘Added’ sugar content refers to free sugar content.

^b^ Free sugar contributed by fruit.

**Table B: Descriptions of yoghurts and custards** **included in choice tasks**

| **Brand** | **Food item** | **Item description** | **High/ low sugar classification** | **Package size (g)** | **Serving size (g)** | **Per 100g** | | | | **Per serving** | | **HSR using total sugars algorithm** | **HSR using added sugars algorithm** |
| --- | --- | --- | --- | --- | --- | --- | --- | --- | --- | --- | --- | --- | --- |
|  |  |  |  |  |  | **Energy (kJ)** | **Fat (g)** | **Total sugar (g)** | **Added sugars (g)** ^a^ | **Added sugar (g)** ^a^ | **Added sugar (nearest teaspoons)** ^a^ |  |  |
| Pauls | Double thick rich chocolate custard | Yoghurt (dessert) | High | 900 | 100 | 637 | 5.9 | 18.5 | 14.0 | 14.0 | 3 | 2.5 | 0.5 |
| Wicked Sister | Premium custard Madagascan vanilla bean | Yoghurt (dessert) | High | 500 | 125 | 574 | 6.4 | 13.4 | 7.5 | 9.5 | 3 | 2.5 | 0.5 |
| Five:am | Honey & cinnamon organic yoghurt | Yoghurt (flavoured) | High | 700 | 100 | 497 | 5.3 | 12.6 | 8.8 | 8.8 | 2 | 3.0 | 1.5 |
| Gippsland Dairy | Strawberry & cream twist yoghurt | Yoghurt (flavoured) | High | 720 | 90 | 618 | 5.7 | 16.5 | 7.6 | 6.8 | 2 | 3.0 | 2.0 |
| Dairy Farmers | Thick & creamy classic vanilla yoghurt | Yoghurt (flavoured) | High | 600 | 150 | 525 | 6.3 | 12.9 | 6.8 | 10.3 | 2 | 2.5 | 2.5 |
| Jalna ^b^ | Yoghurt lactose free whole milk vanilla | Yoghurt (allergen) | Low | 1000 | 100 | 479 | 4.1 | 11.4 | 4.8 | 4.8 | 1 | 4.0 | 3.0 |
| Vaalia | Low fat luscious berries yoghurt | Yoghurt | Low | 900 | 150 | 378 | 2.5 | 9.1 | 4.2 | 6.3 | 1 | 4.0 | 3.0 |
| Tamar Valley | Greek yoghurt | Yoghurt (Greek- plain) | Low | 1000 | 100 | 542 | 9.8 | 5.2 | 0.0 | 0.0 | 0 | 3.0 | 3.5 |
| Danone YoPro ^b^ | Strawberry yoghurt | Yoghurt (flavoured) | Low | 700 | 160 | 243 | 0.3 | 3.3 | 1.8 | 2.9 | 1 | 5.0 | 5.0 |
| Yoplait Forme | Zero French vanilla yoghurt | Yoghurt (vanilla flavoured) | Low | 1000 | 160 | 166 | 0.1 | 4.2 | 0.0 | 0.0 | 0 | 5.0 | 5.0 |

HSR, Health Star Rating

^a^ ‘Added’ sugar content refers to free sugar content.

^b^ Free sugar contributed by fruit.

**Table C: Descriptions of breakfast cereal included in choice tasks**

| **Brand** | **Food item** | **Item description** | **High/ low sugar classification** | **Package size (g)** | **Serving size (g)** | **Per 100g** | | | | **Per serving** | | **HSR using total sugars algorithm** | **HSR using added sugars algorithm** |
| --- | --- | --- | --- | --- | --- | --- | --- | --- | --- | --- | --- | --- | --- |
|  |  |  |  |  |  | **Energy (kJ)** | **Fat (g)** | **Total sugar (g)** | **Added sugars (g)** ^a^ | **Added sugar (g)** ^a^ | **Added sugar (nearest teaspoons)** ^a^ |  |  |
| Nestle | Whole grain energy & fibre milo cereal | Sugary cereal | High | 700 | 30 | 1610 | 4.8 | 26.9 | 22.5 | 6.8 | 2 | 3.5 | ­­1.5 |
| Kellogg's | All-bran high fibre breakfast cereal | Bran cereal | High | 530 | 45 | 1380 | 207 | 16.7 | 16.7 | 7.5 | 2 | 5.0 | 2.5 |
| Sam's pantry | Butter baked oats with roasted almonds toasted muesli | Oats (granola) | High | 475 | 45 | 1770 | 14.9 | 13.6 | 13.6 | 6.1 | 1 | 4.0 | 2.0 |
| Carman's | 5 grain & seed granola pink lady apple & blueberry | Oats (granola) | High | 450 | 45 | 1670 | 13.5 | 14.2 | 9.8 | 4.4 | 1 | 4.5 | 2.5 |
| Kellogg's | Rice bubbles puffed rice breakfast cereal | Whole-grain cereal | High | 705 | 35 | 1630 | 1.1 | 8.5 | 8.5 | 3.0 | 1 | 3.0 | 2.0 |
| Freedom foods | Cereal rice puffs | Whole-grain cereal | Low | 250 | 35 | 1560 | 2.0 | 5.5 | 5.5 | 1.9 | 0 | 4.5 | 4.0 |
| Freedom | Balance buckwheat & quinoa cereal | Whole-grain cereal | Low | 350 | 40 | 1560 | 8.4 | 4.8 | 4.8 | 1.9 | 0 | 5.0 | 4.5 |
| Sanitarium | Weet-bix breakfast cereal | Whole-grain cereal | Low | 575 | 30 | 1490 | 1.3 | 3.3 | 3.3 | 1.0 | 0 | 5.0 | 4.5 |
| Jordans | Low sugar granola blueberry & coconut | Oats (granola) | Low | 500 | 45 | 1890 | 15.2 | 3.2 | 1.0 | 0.4 | 0 | 4.0 | 4.0 |
| Uncle Toby’s | Oats traditional porridge | Oats (rolled) | Low | 500 | 40 | 1600 | 9.2 | 1.0 | 0.0 | 0.0 | 0 | 5.0 | 5.0 |

HSR, Health Star Rating

^a^ ‘Added’ sugar content refers to free sugar content.
