# Supplementary material for: Exploring the effects of added sugar labels on food purchasing behaviour in Australian parents: An online randomised controlled trial
Source: PLoS One. 2022 Aug 25;17(8):e0271435. doi: 10.1371/journal.pone.0271435 (PMC9409597; doi:10.1371/journal.pone.0271435)
Supplement: S6 File — (DOCX) [file pone.0271435.s006.docx]

**S6 File: Participant survey questions**

1. **Are you the main person responsible for buying groceries in your household?**

- Yes- I have sole responsibility
- Yes- I share this responsibility
- No- someone else is responsible

1. **How often would you go to the supermarket or complete an online supermarket order for your household?**

- Twice a week or more
- Once per week
- Less than twice a week but at least once per month
- Less than once per month

1. **Which of the following products have you purchased in person or online from a supermarket in the last month for you or your household?**

*(select all that apply)*

If you purchased all the items below please select *"All of the above"*

If you purchased none of the items below please select *"None of the above"*

- Yoghurt or custard
- Non-alcoholic packaged beverages excluding milk and milk alternatives
- Breakfast cereals
- None of the above
- All of the above

1. **Do you usually shop for other adults or children?**

- Yes
- No

1. **Please indicate how many adults and how many children you usually shop for?**

Please note: Add a zero (0) if the answer is none

- __Adults (18 years or older)
- __Children (less than 18 years)

1. **Which of the following do you purchase at least one month or more for your household from the following options?**

*(select all that apply)*

- Custard
- Flavoured Greek or natural yoghurt
- Plain Greek or natural yoghurt
- None of the above

1. **Which of the following do you purchase at least one month or more for your household from the following options?**

*(select all that apply)*

- Soft drinks (non-diet varieties)
- Iced tea (non-diet varieties)
- Fruit drinks
- Flavoured still or sparkling waters
- diet soft drinks (included low sugar varieties)
- diet iced tea (included low sugar varieties)
- 100% fruit- fruit juice
- Still or sparkling plain bottled water
- None of the above

1. **Which of the following do you purchase at least one month or more for your household from the following options?**

*(select all that apply)*

- Chocolate-flavoured cereals (e.g. coco-pops)
- Toasted muesli or granola (without fruit)
- Cereal marketed to children (e.g. Cheerios, Fruit loops, Nutri-Grain, Rice Bubbles)
- Predominantly oats-based cereal (e.g. rolled oats, Oat Flakes)
- Bran based flakes or biscuits cereals (e.g. All Bran, Guardian, Weet-bix)
- Non bran-based flakes cereal (e.g. corn flakes, special k)
- Cereals with fruit (e.g. Sultana Bran, Just Right, Granola/muesli with fruit)
- None of the above

****Choice tasks ** (see Appendix 1 for sample choice task)**

Participants were randomised to complete one of seven conditions. Each survey condition will include three hypothetical purchasing tasks: one each for non-alcoholic packaged beverages, breakfast cereals, and yoghurts. Each task consisted of selecting between ten different products with varying sugar content, labelled with one of seven labelling options. Participants were given the option to not purchase any of the available products. Participants were asked to confirm if they agree to leave the store without make a purchase of an item they intended to buy or if this was an error. When this was an error, participants were reassigned to the previous question and asked to select a product.

**Post choice task questions:**

1. **Thinking about the choices you just made, which of the following did you consider in making your decision?**

*(select all that apply)*

- Taste
- Familiarity with product
- Healthiness of the item
- Family preferences
- Time of day
- Other _____

1. **Please select all the following statements you agree with**

*[image of full-sugar Coca Cola bottle; removed from publication for copyright reasons]*

**Drinking the above item would…**

*(select all that apply)*

- …lead to weight gain
- …increase my risk of heart disease
- …have no impact on my health
- …increase my risk of diabetes
- …help me live a healthier life
- …increase my risk of dental decay
- …increase my productivity
- …make social occasions more enjoyable

1. **If high sugar products carried this health message (which provided the number of teaspoons of added sugar in product) on the front of package, what would be your most likely response?**

*(select all that apply)*


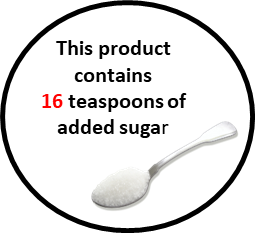


- Buy a smaller package size of the product (if available)
- Buy the product less frequently
- Find a lower sugar alternative
- Find a no added sugar alternative
- Stop purchasing this item
- No change in purchasing
- Unsure what I would do
- Other (please specify)____

1. **If high sugar products carried this health symbol (indicated the healthiness of the item, where 0.5= least healthy and 5 stars= most healthy) on the front of package, what would be your most likely response?**

*(select all that apply)*


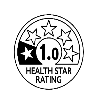
 *[Health Star Rating trademarks are owned by the Commonwealth of Australia. Further information on the Health Star Rating can be found at www.healthstarrating.gov.au.]*

- Buy a smaller package size of the product (if available)
- Buy the product less frequently
- Find a lower sugar alternative
- Find a no added sugar alternative
- Stop purchasing this item
- No change in purchasing
- Unsure what I would do
- Other (please specify)____

1. **What is the minimum number of health stars a product would need for you to consider it to be "healthy"?**

- 0.5
- 1.0
- 1.5
- 2.0
- 2.5
- 3.0
- 3.5
- 4.0
- 4.5
- 5.0

1. **If high sugar products carried this advisory warning on the front of package, what would be your most likely response?**

*(select all that apply)*

**HIGH IN SUGAR**

- Buy a smaller package size of the product (if available)
- Buy the product less frequently
- Find a lower sugar alternative
- Find a no added sugar alternative
- Stop purchasing this item
- No change in purchasing
- Unsure what I would do
- Other (please specify)____

1. **If products highlighted which ingredients contained added sugar on the ingredients list on the back of the package, what would be your most likely response?**

*(select all that apply)*

Ingredient list: Carbonated water, high fructose corn syrup*, citrate acid, natural flavours, sodium citrate, sodium benzoate

*sugar-based ingredients

- Buy a smaller package size of the product (if available)
- Buy the product less frequently
- Find a lower sugar alternative
- Find a no added sugar alternative
- Stop purchasing this item
- No change in purchasing
- Unsure what I would do
- Other (please specify)____

1. **If products highlighted added sugar in the nutrition information panel on the back of the package, what would be your most likely response?**

*(select all that apply)*

| **NUTRITION INFORMAITON** |  | |
| --- | --- | --- |
| **Servings per package: 8**  **Serving size: 250ml** | | |
| **Av. Quantity** | **Per Serving** | **Per 100ml** |
| **Energy** | **450KJ** | **180KJ** |
| **Protein** | **0.1g** | **0.05g** |
| **Fat total** | **0g** | **0g** |
| - **Saturated** | **0g** | **0g** |
| **Carbohydrates** | **26.5g** | **10.6g** |
| - **Total sugars** | **26.5g** | **10.6g** |
| - **Added sugars** | **26.5g** | **10.6g** |
| **Dietary Fibre** | **0g** | **0g** |
| **Sodium** | **25mg** | **0.5mg** |

- Buy a smaller package size of the product (if available)
- Buy the product less frequently
- Find a lower sugar alternative
- Find a no added sugar alternative
- Stop purchasing this item
- No change in purchasing
- Unsure what I would do
- Other (please specify) ___________

1. **Indicate how strongly you agree with the following statements**

|  | Strongly agree | Agree | Neither agree nor disagree | Disagree | Strongly disagree |
| --- | --- | --- | --- | --- | --- |
| Government should require stricter standards to ensure that food corporations clearly identify high sugar levels in products. |  |  |  |  |  |
| Consumers need more information to make informed decisions about healthy food products |  |  |  |  |  |
| We need to set higher standards for how the food industry labels the food we eat |  |  |  |  |  |
| We need more nutrition information on food labels so consumers can make the right choices |  |  |  |  |  |

1. **What is your gender?**

- Woman
- Man
- Non-binary/ gender diverse
- My gender identity is not listed
- Prefer not to say

1. **What is your post code?**

**_______**

1. **What is your age?**

- 18 to 25 years
- 26 to 35 years
- 36 to 45 years
- 46 to 55 years
- 56 to 65 years
- 66 to 75 years
- 76 years or older

1. **Is English in your first language?**

- Yes
- No

1. **Are you of Aboriginal or Torres Strait Islander origin?**

- No
- Yes, Aboriginal
- Yes, Torres Strait Islander
- Yes, both Aboriginal and Torres Strait Islander

1. **What is the highest level of education you have completed?**

- Primary school or below
- High school year 11 or below
- Completed high school (year 12, form 6 HSC)
- TAFE or Trade Certificate or Diploma
- Undergraduate university degree (i.e., bachelor, diploma)
- Postgraduate university degree (i.e., postgraduate diploma, masters, PhD)

1. **Before tax is taken out, which of the following ranges best describes your household’s income over the last 12 months? Please include income from all sources, including wages, investments and government pensions and benefits.**

- Less than or equal to $24,999
- $25,000 to $38,999
- $39,000 to $52,999
- $53,000 to $69,999
- $70,000 to $88,999
- $89,000 to $109,999
- $110,000 to $134,999
- $135,000 to $168,999
- $169,000 to $222,999
- More than or equal to $223,000

1. **Which of these best describes your current employment status?**

o Employed full-time

o Employed part-time

o Self-employed/ freelancer

o Casual

o Unemployed

o Unable to work due to COVID-19

o Retired

o Not employed – a student

o Not employed – engaged in home duties

o Not employed – unable to work

o Other (please specify)____

1. **Do you know your current height (without shoes)? (if you select yes you will be asked to provide your height)**

- Yes
- No
- I would prefer not to answer

If yes:

1. **Please enter the parameters you would like to enter your height in.**

- centimeters
- feet-inches

1. **How tall are you (without shoes)? You do not need to provide units.**

- centimeters ________________
- feet-inches _______ - ________

1. **Do you know your current weight in light clothing? (if you select yes you will be asked to provide your height)**

- Yes
- No
- I would prefer not to answer

If yes:

1. **Please enter the parameters you would like to enter your weight in.**

- kilograms
- stone-pounds

1. **What is your current weight in light clothing?**

- kilograms _________________

1. **What is your current weight in light clothing?**

- stone - pounds ______ - ______

1. **What best describes your current goals for your weight?**

- I am trying to lose weight
- I am trying to gain weight
- I am trying to maintain my weight (neither gain nor lose)
- I do not have a goal for my weight

1. **In the past week, on how many days have you done a total of 30 minutes or more of physical activity, which was enough to raise your breathing rate? This may include organized sport, exercise, or brisk walking or cycling for recreation or to get to and from places but should not include housework or physical activity that may be part of your job. Provide an answer between 0 and 7 days.**

- ______ days

1. **When you complete your supermarket shop, who are you usually buying food items for?** Select all that apply

- Myself
- One or more children younger than 18 years in my household
- Another adult over the age of 18 years in my household
- Other (please specify)____

1. **Which of these is the main source of food you purchase for your household?**

- Convenience store
- Supermarket
- Greengrocer or other specialty store
- Fast food/restaurant/café
- Online
- Meal kit delivery
- Other (please specify)____

**Thank you for your time and assisting us with this survey. When you click 'Submit' the survey will be complete. If you would like to make any further comments, please enter them below.**
