# Supplementary material for: Exploring the effects of added sugar labels on food purchasing behaviour in Australian parents: An online randomised controlled trial
Source: PLoS One. 2022 Aug 25;17(8):e0271435. doi: 10.1371/journal.pone.0271435 (PMC9409597; doi:10.1371/journal.pone.0271435)
Supplement: S7 File — (DOCX) [file pone.0271435.s007.docx]

S7 File. Detailed results

**Table A: Demographic characteristics by inclusion in analysis of those randomised to a labelling condition**

| **Characteristic** | **n (%)** | | | **p-value** |
| --- | --- | --- | --- | --- |
|  | **Total (n=2825)** | **Included**  **(n=2582)** | **Excluded**  **(n=243)** |  |
| **Gender (base: Man)** | | | | |
| Woman | 1570 (56.6) | 1471 (57.8) | 99 (43.2) | <0.001 |
| **Age (years) (base: 18 to 35 years)** | | | |  |
| 36 years or older | 1790 (64.0) | 1641 (64.0) | 149 (64.5) | 0.867 |
| **Educational attainment (base: less than university)** | | | |  |
| University | 1533 (54.8) | 1394 (54.3) | 139 (60.2) | 0.087 |
| **Equivalised household income per week (AUD)** ^a^ **(base: <$899)** | | | |  |
| ≥$899 | 1145 (40.5) | 999 (38.7) | 146 (60.1) | <0.001 |
| **Employment (base: employed less than full-time)** | | | |  |
| Employed full-time | 1514 (54.1) | 1369 (53.4) | 145 (62.8) | 0.006 |
| **Socioeconomic Index for Areas (SEIFA) (base: low)** | | | |  |
| High | 1851 (66.5) | 1693 (66.3) | 158 (68.7) | 0.464 |
| **Weight loss goals (base: Trying to maintain weight or do not have a goal)** | | | |  |
| Trying to lose weight | 1609 (60.7) | 1471 (60.1) | 138 (68.0) | 0.027 |
| **Body Mass Index (base: BMI <25 kg/m^2^)** ^b^ | | | | |
| **BMI ≥25 kg/m^2^** | 1046 (54.3) | 953 (54.4) | 93 (53.8) | 0.533 |

^a^ Adjusted for number of adults and children in a household.

^b^ 899 participants did not know or elected not to disclose their height and/or weight

**Table B: Demographic characteristics by regular sugar purchasing classification**

| **Characteristic** | **n (%)** | | | **p-value** |
| --- | --- | --- | --- | --- |
|  | **Total (n=2582)** | **Usually purchases low sugar products (n=1260)** | **Usually purchases high sugar products (n=1322)** |  |
| **Gender (base: Man)** | | | | |
| Woman | 1471 (57.8) | 772 (62.26) | 699 (53.56) | <0.001 |
| **Age (years) (base: 18 to 35 years)** | | | |  |
| 36 years or older | 1641 (63.95) | 832 (66.83) | 809 (61.24) | 0.003 |
| **Educational attainment (base: less than university)** | | | |  |
| University | 1394 (54.19) | 663 (53.25) | 731 (55.34) | 0.290 |
| **Equivalised household income per week (AUD)** ^a^ **(base: <$899)** | | | |  |
| ≥$899 | 999 (38.69) | 495 (39.29) | 504 (38.12) | 0.545 |
| **Employment (base: employed less than full-time)** | | | |  |
| Employed full-time | 1369 (53.35) | 584 (46.91) | 785 (59.42) | <0.001 |
| **Socioeconomic Index for Areas (SEIFA) (base: high socioeconomic disadvantage)** | | | |  |
| Low disadvantage | 1693 (66.31) | 823 (66.42) | 870 (66.21) | 0.909 |
| **Weight loss goals (base: Trying to maintain weight or do not have a goal)** | | | |  |
| Trying to lose weight | 1471 (60.07) | 733 (59.07) | 738 (61.09) | 0.306 |
| **Body Mass Index (base: BMI <25 kg/m^2^)** ^b^ | | | | |
| **BMI ≥25 kg/m^2^** | 1753 (54.36) | 516 (57.14) | 437 (51.41) | 0.016 |

^a^ Adjusted for number of adults and children in a household.

^b^ 899 participants did not know or elected not to disclose their height and/or weight

**Table C: Effect of added sugar labels on odds of selecting a high sugar product, overall (n=2582 participants)**

| **Label** | **Odds Ratio ^a^** | **95%CI** | **SE** | **P value** |
| --- | --- | --- | --- | --- |
| Constant ^b^ | 0.73 | 0.64, 0.82 | 0.05 | <0.001 |
| B: Nutrition Information Panel with Added Sugar | 1.00 | 0.83, 1.20 | 0.09 | 0.993 |
| C: Teaspoons of Sugar | 0.94 | 0.80, 1.11 | 0.08 | 0.489 |
| D: Warning | 1.10 | 0.93, 1.30 | 0.10 | 0.264 |
| E: HSR with Total Sugar | 1.01 | 0.85, 1.21 | 0.09 | 0.895 |
| F: HSR with Added Sugar | 1.09 | 0.92, 1.30 | 0.10 | 0.319 |
| G: Sugar in the Ingredients List | 1.01 | 0.85, 1.21 | 0.09 | 0.886 |

HSR, Health Star Rating. Wald chi2(6) = 3.86; Prob>chi2 = 0.6957

**^a^** Overall analysis was adjusted for clustering to take account of repeated participants between categories.

^b^ Reference: A: Control

**Table D: Effect of added sugar labels on odds of selecting a high sugar product, beverages (n=2582 participants)**

| **Label** | **Odds Ratio** | **95%CI** | **SE** | **P value** |
| --- | --- | --- | --- | --- |
| Constant ^a^ | 1.40 | 1.14, 1.72 | 0.15 | 0.002 |
| B: Nutrition Information Panel with Added Sugar | 0.61 | 0.46, 0.82 | 0.09 | 0.001 |
| C: Teaspoons of Sugar | 0.73 | 0.54, 0.97 | 0.11 | 0.032 |
| D: Warning | 0.88 | 0.66, 1.18 | 0.13 | 0.402 |
| E: HSR with Total Sugar | 0.76 | 0.57, 1.02 | 0.11 | 0.069 |
| F: HSR with Added Sugar | 0.84 | 0.63, 1.12 | 0.12 | 0.228 |
| G: Sugar in the Ingredients List | 0.81 | 0.61, 1.09 | 0.12 | 0.164 |

HSR, Health Star Rating. Likelihood Ratio chi squared (6) = 13.09; Prob > chi2 = 0.0416

^a^ Reference: A: Control

**Table E: Effect of added sugar labels on odds of selecting a high sugar product, breakfast cereals (n=2582 participants)**

| **Label** | **Odds Ratio** | **95%CI** | **SE** | **P value** |
| --- | --- | --- | --- | --- |
| Constant ^a^ | 0.49 | 0.40, 0.00 | 0.05 | <0.001 |
| B: Nutrition Information Panel with Added Sugar | 1.17 | 0.86, 1.59 | 0.18 | 0.311 |
| C: Teaspoons of Sugar | 0.99 | 0.73, 1.35 | 0.16 | 0.959 |
| D: Warning | 1.15 | 0.85, 1.56 | 0.18 | 0.369 |
| E: HSR with Total Sugar | 1.22 | 0.90, 1.65 | 0.19 | 0.199 |
| F: HSR with Added Sugar | 1.35 | 1.00, 1.82 | 0.21 | 0.051 |
| G: Sugar in the Ingredients List | 1.19 | 0.88, 1.60 | 0.18 | 0.272 |

HSR, Health Star Rating. Likelihood Ratio chi squared (6) = 6.06; Prob > chi2 = 0.4161

^a^ Reference: A: Control

**Table F: Effect of added sugar labels on odds of selecting a high sugar product, yoghurts (n=2582 participants)**

| **Label** | **Odds Ratio** | **95%CI** | **SE** | **P value** |
| --- | --- | --- | --- | --- |
| Constant ^a^ | 0.54 | 0.43, 0.66 | 0.06 | <0.001 |
| B: Nutrition Information Panel with Added Sugar | 1.40 | 1.04, 1.89 | 0.21 | 0.027 |
| C: Teaspoons of Sugar | 1.17 | 0.87, 1.58 | 0.18 | 0.310 |
| D: Warning | 1.30 | 0.96, 1.75 | 0.20 | 0.088 |
| E: HSR with Total Sugar | 1.13 | 0.84, 1.53 | 0.17 | 0.415 |
| F: HSR with Added Sugar | 1.17 | 0.87, 1.58 | 0.18 | 0.302 |
| G: Sugar in the Ingredients List | 1.13 | 0.83, 1.52 | 0.17 | 0.436 |

HSR, Health Star Rating. Likelihood Ratio chi squared (6) = 6.03; Prob > chi2 = 0.4193

^a^ Reference: A: Control

**Table G: Effect of added sugar labels on added sugar content of purchases, overall ^a^ (n=2582 participants)**

| **Label** | **Change in added sugar g/100g** | | **SE** | **P value** |
| --- | --- | --- | --- | --- |
|  | **Coefficient** | **95%CI** |  |  |
| Constant ^b^ | 6.44 | 6.02, 6.85 | 0.21 | <0.001 |
| B: Nutrition Information Panel with Added Sugar | -0.54 | -1.14, 0.06 | 0.31 | 0.079 |
| C: Teaspoons of Sugar | -0.46 | -1.07, 0.14 | 0.31 | 0.135 |
| D: Warning | 0.04 | -0.55, 0.64 | 0.30 | 0.890 |
| E: HSR with Total Sugar | -0.37 | -0.98, 0.24 | 0.31 | 0.234 |
| F: HSR with Added Sugar | 0.03 | -0.57, 0.63 | 0.31 | 0.930 |
| G: Sugar in the Ingredients List | -0.24 | -0.84, 0.36 | 0.31 | 0.433 |

HSR, Health Star Rating. F(6, 2576) = 1.25; Prob > F = 0.2786

**^a^** Overall analysis was adjusted for clustering to take account of repeated participants between categories

**^b^** Reference: A: Control

**Table H: Effect of added sugar labels on added sugar content of purchases, beverages (n=2582 participants)**

| **Label** | **Change in added sugar g/100mL** | | **SE** | **P value** |
| --- | --- | --- | --- | --- |
|  | **Coefficient** | **95%CI** |  |  |
| Constant ^a^ | 12.42 | 11.32, 13.53 | 0.56 | <0.001 |
| B: Nutrition Information Panel with Added Sugar | -2.35 | -3.92, -0.78 | 0.80 | 0.003 |
| C: Teaspoons of Sugar | -1.68 | -3.24, -0.11 | 0.80 | 0.036 |
| D: Warning | -0.46 | -2.02, 1.10 | 0.79 | 0.562 |
| E: HSR with Total Sugar | -1.24 | -2.81, 0.33 | 0.80 | 0.121 |
| F: HSR with Added Sugar | -0.62 | -2.19, 0.94 | 0.80 | 0.434 |
| G: Sugar in the Ingredients List | -1.15 | -2.71, 0.40 | 0.79 | 0.145 |

HSR, Health Star Rating. F(6, 2446) = 1.96; Prob > F = 0.0680

**^a^** Reference: A: Control

**Table I: Effect of added sugar labels on added sugar content of purchases, breakfast cereals (n=2582 participants)**

| **Label** | **Change in added sugar g/100g** | | **SE** | **P value** |
| --- | --- | --- | --- | --- |
|  | **Coefficient** | **95%CI** |  |  |
| Constant ^a^ | 2.29 | 2.02, 2.55 | 0.13 | <0.001 |
| B: Nutrition Information Panel with Added Sugar | 0.17 | -0.21, 0.54 | 0.19 | 0.381 |
| C: Teaspoons of Sugar | 0.10 | -0.27, 0.47 | 0.19 | 0.602 |
| D: Warning | 0.30 | -0.07, 0.67 | 0.19 | 0.110 |
| E: HSR with Total Sugar | 0.24 | -0.13, 0.61 | 0.19 | 0.199 |
| F: HSR with Added Sugar | 0.38 | 0.01, 0.75 | 0.19 | 0.046 |
| G: Sugar in the Ingredients List | 0.28 | -0.08, 0.65 | 0.19 | 0.131 |

HSR, Health Star Rating. F(6, 2446) = 1.96; Prob > F = 0.0680

**^a^** Reference: A: Control

**Table J: Effect of added sugar labels on added sugar content of purchases, yoghurts (n=2582 participants)**

| **Label** | **Change in added sugar g/100g** | | **SE** | **P value** |
| --- | --- | --- | --- | --- |
|  | **Coefficient** | **95%CI** |  |  |
| Constant ^a^ | 4.70 | 4.23, 5.18 | 0.24 | <0.001 |
| B: Nutrition Information Panel with Added Sugar | 0.50 | -0.17, 1.17 | 0.34 | 0.143 |
| C: Teaspoons of Sugar | 0.26 | -0.41, 0.93 | 0.34 | 0.455 |
| D: Warning | 0.26 | -0.41, 0.93 | 0.34 | 0.440 |
| E: HSR with Total Sugar | -0.03 | -0.70, 0.64 | 0.34 | 0.936 |
| F: HSR with Added Sugar | 0.35 | -0.32, 1.02 | 0.34 | 0.302 |
| G: Sugar in the Ingredients List | 0.10 | -0.57, 0.76 | 0.34 | 0.775 |

HSR, Health Star Rating. F(6, 2458) = 0.64; Prob > F = 0.7019

**^a^** Reference: A: Control

**Table K: Effect of added sugar labels on Health Star Rating** ^a^ **of purchases, overall ^b^ (n=2582 participants)**

| **Label** | **Estimated change in Health Star Rating ^a^** | | **SE** | **P value** |
| --- | --- | --- | --- | --- |
|  | **Coefficient** | **95%CI** |  |  |
| Constant ^c^ | 3.58 | 3.52, 3.65 | 0.03 | <0.001 |
| B: Nutrition Information Panel with Added Sugar | 0.05 | -0.04, 0.15 | 0.05 | 0.238 |
| C: Teaspoons of Sugar | 0.05 | -0.04, 0.13 | 0.05 | 0.324 |
| D: Warning | -0.01 | -0.10, 0.08 | 0.05 | 0.818 |
| E: HSR with Total Sugar ^a^ | 0.05 | -0.04, 0.15 | 0.05 | 0.273 |
| F: HSR with Added Sugar | -0.02 | -0.12, 0.07 | 0.05 | 0.615 |
| G: Sugar in the Ingredients List | -0.03 | -0.13, 0.06 | 0.05 | 0.473 |

HSR, Health Star Rating. F(6, 2576) = 1.28; Prob > F = 0.2620

^a^ Proposed updated Health Star Rating based on recommendations from 5-year review including stricter penalties for total sugar in algorithm

**^b^** Overall analysis was adjusted for clustering to take account of repeated participants between categories

^c^ Reference: A: Control

**Table L: Effect of added sugar labels on Health Star Rating** ^a^ **of purchases, beverages (n=2582 participants)**

| **Label** | **Estimated change in Health Star Rating ^a^** | | **SE** | **P value** |
| --- | --- | --- | --- | --- |
|  | **Coefficient** | **95%CI** |  |  |
| Constant ^b^ | 3.58 | 3.52, 3.65 | 0.03 | <0.001 |
| B: Nutrition Information Panel with Added Sugar | 0.05 | -0.04, 0.15 | 0.05 | 0.238 |
| C: Teaspoons of Sugar | 0.05 | -0.04, 0.13 | 0.05 | 0.324 |
| D: Warning | -0.01 | -0.10, 0.08 | 0.05 | 0.818 |
| E: HSR with Total Sugar ^a^ | 0.05 | -0.04, 0.15 | 0.05 | 0.273 |
| F: HSR with Added Sugar | -0.02 | -0.12, 0.07 | 0.05 | 0.615 |
| G: Sugar in the Ingredients List | -0.03 | -0.13, 0.06 | 0.05 | 0.473 |

HSR, Health Star Rating. F(6, 2576) = 1.28; Prob > F = 0.2620

^a^ Proposed updated Health Star Rating based on recommendations from 5-year review including stricter penalties for total sugar in algorithm

^b^ Reference: A: Control

**Table M: Effect of added sugar labels on Health Star Rating** ^a^ **of purchases, breakfast cereals (n=2582 participants)**

| **Label** | **Estimated change in Health Star Rating ^a^** | | **SE** | **P value** |
| --- | --- | --- | --- | --- |
|  | **Coefficient** | **95%CI** |  |  |
| Constant ^b^ | 4.57 | 4.50, 4.63 | 0.03 | <0.001 |
| B: Nutrition Information Panel with Added Sugar | -0.08 | -0.17, 0.02 | 0.05 | 0.118 |
| C: Teaspoons of Sugar | -0.03 | -0.12, 0.07 | 0.05 | 0.600 |
| D: Warning | -0.01 | -0.11, 0.08 | 0.05 | 0.770 |
| E: HSR with Total Sugar ^a^ | -0.08 | -0.18, 0.01 | 0.05 | 0.098 |
| F: HSR with Added Sugar | -0.09 | -0.19, 0.01 | 0.05 | 0.063 |
| G: Sugar in the Ingredients List | -0.03 | -0.12, 0.07 | 0.05 | 0.561 |

HSR, Health Star Rating. F(6, 2517) = 1.12; Prob > F = 0.3475

^a^ Proposed updated Health Star Rating based on recommendations from 5-year review including stricter penalties for total sugar in algorithm

^b^ Reference: A: Control

**Table N: Effect of added sugar labels on Health Star Rating** ^a^ **of purchases, yoghurts (n=2582 participants)**

| **Label** | **Estimated change in Health Star Rating ^a^** | | **SE** | **P value** |
| --- | --- | --- | --- | --- |
|  | **Coefficient** | **95%CI** |  |  |
| Constant ^b^ | 3.41 | 3.31, 3.51 | 0.05 | <0.001 |
| B: Nutrition Information Panel with Added Sugar | -0.02 | -0.16, 0.11 | 0.07 | 0.726 |
| C: Teaspoons of Sugar | -0.05 | -0.19, 0.09 | 0.07 | 0.465 |
| D: Warning | -0.05 | -0.19, 0.09 | 0.07 | 0.490 |
| E: HSR with Total Sugar ^a^ | 0.08 | -0.06, 0.22 | 0.07 | 0.278 |
| F: HSR with Added Sugar | 0.03 | -0.11, 0.17 | 0.07 | 0.670 |
| G: Sugar in the Ingredients List | -0.18 | -0.32, -0.04 | 0.07 | 0.012 |

HSR, Health Star Rating. F(6, 2458) = 2.59; Prob > F = 0.0167

^a^ Proposed updated Health Star Rating based on recommendations from 5-year review including stricter penalties for total sugar in algorithm

^b^ Reference: A: Control

**Table O: Participant stated most likely purchasing response to introduction of added sugar labelling policy options (n=2582 participants)**

| **Most likely response:** |  |  | **n (%)** |  |  |
| --- | --- | --- | --- | --- | --- |
|  | **Nutrition Information Panel with Added Sugar (n= 2576)** | **Warning label**  **(n= 2576)** | **Teaspoons of Sugar (n=2579)** | **Sugar in Ingredients List**  **(n=2572)** | **Health Star Rating (n=2576)** |
| Buy a smaller version of the product | 85 (3.31) | 89 (3.45) | 104 (4.03) | 70 (2.72) | 86 (3.34) |
| Buy the product less frequently | 360 (14.0) | 440 (17.1) | 481 (18.7) | 366 (14.2) | 510 (19.8) |
| Find a lower sugar alternative | 651 (25.3) | 575 (22.3) | 477 (18.5) | 502 (19.5) | 413 (16.0) |
| Find a no added sugar alternative | 444 (17.3) | 368 (14.3) | 503 (19.5) | 409 (15.9) | 344 (13.4) |
| Stop purchasing this item | 322 (12.5) | 658 (25.5) | 659 (25.6) | 368 (14.3) | 658 (25.5) |
| No change in purchasing | 501 (19.5) | 292 (11.3) | 237 (9.19) | 564 (21.9) | 370 (14.4) |
| Unsure what I would do | 192 (7.47) | 131 (5.09) | 90 (3.49) | 269 (10.5) | 163 (6.33) |
| Other (please specify) | 15 (0.58) | 23 (0.89) | 28 (1.09) | 24 (0.93) | 32 (1.24) |

**Table P: Participant agreement with different framing of added sugar labelling policy (n=2569 participants)**

| **Sugar labelling policy framing** | **n (%)** | | | | |
| --- | --- | --- | --- | --- | --- |
|  | **Strongly disagree** | **Disagree** | **Neither agree nor disagree** | **Agree** | **Strongly agree** |
| Government should require stricter standards to ensure that food corporations clearly identify high sugar levels in products | 72 (2.80) | 77 (3.00) | 314 (12.2) | 1025 (39.9) | 1081 (42.1) |
| Consumers need more information to make informed decisions about healthy food products | 65 (2.53) | 105 (4.09) | 305 (11.9) | 1097 (42.7) | 997 (38.8) |
| We need to set higher standards for how the food industry labels the food we eat | 64 (2.49) | 63 (2.45) | 271 (10.6) | 1020 (39.7) | 1151 (44.8) |
| We need more nutrition information on food labels so consumers can make the right choices | 59 (2.30) | 65 (2.53) | 325 (12.7) | 1046 (40.7) | 1074 (41.8) |
